# Hydrodynamic effects of underground dams on seawater–freshwater mixing zone in sloping beach coastal aquifers

**DOI:** 10.1038/s41598-026-60176-8

**Published:** 2026-07-15

**Authors:** Asaad M. Armanuos, Martina Zeleňáková

**Affiliations:** 1 Irrigation and Hydraulics Engineering Department, Faculty of Engineering, Tanta University, Tanta, Egypt; 2 Institute of Environmental Engineering, Faculty of Civil Engineering, Technical University of Košice, 04200 Košice, Slovakia

**Keywords:** Underground dam, Mixing zone, Seawater intrusion, Coastal aquifers, Sloped beach, Environmental sciences, Hydrology, Solid Earth sciences

## Abstract

Seawater intrusion (SWI) is a continual challenge that threatens the sustainability of coastal groundwater resources. The behaviour of the seawater-freshwater mixing zone plays a critical role in controlling groundwater quality and solute transport; however, the combined impact of underground dams and sloping beach geometries on seawater-freshwater mixing-zone dynamics remains insufficiently understood. This study investigates the hydrodynamic response of SWI in sloping beach coastal aquifers using numerical simulations with beach inclination angles of 0°, 15°, 30°, and 45°, and under various underground dam heights (H_d_). The outcomes indicate that the mixing zone initially expands during the transient stage before shrinking after the seawater intrusion wedge reaches the underground dam. Compared with no-dam conditions, underground dams reduced the final steady-state mixing-zone width and area, while only slightly affecting subsurface groundwater discharge and seawater intrusion length. Increasing dam height produced a narrower central mixing zone and lower subsurface groundwater discharge, whereas its effect on the bottom mixing-zone width was limited. In contrast, increasing the beach inclination angle significantly enhanced seawater intrusion and widened the mixing zone, particularly under steep slopes. Inclined beaches also delayed the time required for the seawater intrusion wedge to reach the dam because of the longer seawater intrusion pathway. The findings demonstrate that aquifer geometry and underground dam configuration strongly control the temporal evolution of seawater intrusion. Proper optimisation of underground dam height is therefore essential for reducing salinity expansion and improving groundwater protection in coastal aquifers. The study provides practical guidance for the design of physical barriers and sustainable groundwater management in vulnerable coastal regions.

## Introduction

Seawater intrusion (SWI) occurs naturally in relation to the salinity differences between freshwater and seawater^1^. However, because of this natural equilibrium being upset in coastal aquifers, the environment and the groundwater quality are disturbed^2^. According to Todd^3^, several strategies have been attempted to control SWI in coastal aquifers. Some of these strategies (mixed barriers) include pumping seawater along the shoreline (abstraction or negative barriers), moving extraction wells, reducing abstraction well rates, using physical surface or underground barriers and employing natural or artificial groundwater recharge (pressure or positive barriers). In coastal aquifers, subsurface physical barriers, such as cutoffs and underground dams, are a common method of preventing seawater invasion.

Subsurface physical barriers for saltwater intrusion control mainly, underground dams and cutoff walls, have been widely investigated, with underground dams being the most applied technique. Overall findings display that these subsurface barriers can modify the groundwater flow and reduce the seawater intrusion, but their efficiency is controlled by key hydrogeological and design factors such as geometry, hydraulic properties, and site conditions^4–8^, (Senthilkumar and Elango, 2011). Currently, there are three types of physical barriers: semi-permeable subsurface barriers (SPSB), cutoff walls, and underground dams^4^. In the global field of avoiding SWI, underground dams are probably the most used type of wall^5^. Jamali et al.^6^ investigated a siting solution for underground dams using a Geographical Information System model connected to a groundwater balancing model. The impact of underground dams on the flow of groundwater in the Indian Palar River basin was investigated by Senthilkumar and Elango (2011) using the groundwater simulation software MODFLOW. According to the model, following the construction of underground dams, the groundwater level in the downstream would drop by 0.1–0.2 m, and the groundwater levels in the upstream would increase by 0.1–0.3 m, with a range of the impact of roughly 1.5–2 km upstream. Abdoulhalik and Ahmed^7^ investigated the cutoff wall effectiveness for SWI management in stratified aquifers using laboratory testing and SEAWAT numerical modelling. To increase the effectiveness of SWI mitigation, they mixed the usage of cutoff walls with underground dams for the initial time^8^. According to Kaleris and Ziogas^4^, the depth of a cutoff wall, the aquifer hydraulic conductivity, the distance between the cutoff wall and the shore, the velocity of the groundwater and aquifer anisotropy all affect how effective underground dams mitigate SWI.

Underground dams can reduce the extent of seawater intrusion, but their effectiveness depends strongly on design and site conditions, and improper configurations may even worsen salt accumulation between the sea boundary and dam location. Studies consistently demonstrated that the optimal dam height and position, along with favorable hydraulic settings, improve fresh groundwater flow and enhance the salt removal from coastal aquifers^9,10^. Underground dams can help reduce seawater invasion, but they can also unintentionally cause inland contaminants to build up. Using flow-tank tests and numerical modelling, Chang et al.^9^ assessed how dam height, position and groundwater head changes affected the flow of groundwater and seawater intrusion wedge size. The outcomes showed that the ideal dam height (38% of aquifer saturated thickness) and position (6.3% of aquifer length from the seawater boundary) increase freshwater discharge and reduce seawater invasion. Since very tall dams worsen seawater intrusion, future studies will include field testing and account for tidal effects. Underground dams and other seawater intrusion preventative measures could remain behind residual seawater inland, necessitating efficient removal techniques. Yin et. al.^10^ investigated the effects of hydraulic controls, aquifer characteristics and dam design on salt removal using lab experiments and simulations. Shorter dams and adjacent freshwater recharge/discharge increase the effectiveness of salt removal. The findings stress how crucial dam placement and height are for quickening aquifer desalination.

Recent studies investigate that saltwater intrusion in stratified coastal aquifers is mainly governed by permeability, layering, and hydraulic stresses for example pumping and sea-level rise. Overall, the heterogeneous conditions and higher permeability intensify seawater intrusion, however subsurface and mixed barriers can successfully limit its spread and preserve freshwater, with performance strongly influenced by aquifer structure and subsurface barrier design^11–13^. Sharma et al.^12^ examined the saltwater intrusion in the coastal stratified aquifers influenced by sea-level rise and excessive groundwater abstraction. The study experimentally investigated the seawater intrusion patterns in coastal aquifers with parallel and perpendicular stratification considering sloping beach conditions. Outcomes confirmed that higher permeability values and layer configuration substantially enhance the depth, and the extent of saltwater intrusion. In addition, the seawater intrusion remains limited within the permeable layers until reaching the impermeable barriers, whereas the larger openings increase the seawater intrusion spread. Sharma and Chakma^11,13^ studied the saltwater intrusion in the stratified coastal aquifers affected by the excessive freshwater extraction utilizing a laboratory-scale experimental model. The study examined the impact of inclined ocean boundaries and mixed barrier remediation techniques on seawater intrusion behavior under various stratification conditions. The results demonstrated that mixed barriers substantially diminished saltwater intrusion extent and delayed saline progression, particularly in the parallel stratified formations. The research also revealed that permeability changes and interaction gap openings strongly control seawater intrusion patterns, emphasizing the efficiency of mixed barriers for heterogeneous coastal aquifers.

Sharma and Chakma^11,13^ investigated the saltwater intrusion behavior in heterogeneous coastal aquifers under sloping boundaries and assessed the performance of subsurface barriers as a remediation measure. The study experimentally confirmed that barrier installation preserved approximately 39% of fresh groundwater from salinity contamination. Results showed that homogeneous aquifers experienced faster seawater advancement, however heterogeneous formations slowed intrusion because of lower permeability conditions. The analysis further confirmed that barrier height strongly impacts remediation effectiveness, with taller barriers supporting greater intrusion control and diminished toe-length expansion.

Using 3D numerical simulations, Wu et al.^14^ assessed the effectiveness of shore-parallel subsurface barriers in avoiding saltwater invasion. In barrier-only and barrier-well situations, they compared underground dams, cutoff walls and fully penetrating barriers. Cutoff walls functioned best along the coast in barrier-only arrangements, whereas underground dams placed 300 m inland, in conjunction with abstraction wells, functioned best. Effectiveness has been shown to be significantly impacted by barrier length, with fully penetrating barriers typically having the best results. Recent experimental and numerical studies have significantly expanded our understanding of SWI control mechanisms. Emara et al.^15,16^ demonstrated that the ideal wall depth and inclination can reduce SWI penetration by up to 65% in experimental tests and numerical simulations of the effects of aquifer heterogeneity and inclined cutoff walls on seawater wedge dynamics. Armanuos et al.^17,18^ used the SEAWAT model to study the effects of physical barriers and coupled recharge wells in sloping coastal aquifers. They found that combining barrier and recharge techniques results in a more efficient SWI retreat than either strategy alone. Furthermore, Armanuos et al.^19^ examined the effect of groundwater abstraction through fractured dams on SWI management, emphasising the importance of considering well placement, abstraction well rates and fracture features when developing control methods. The causes and effects of double cutoff walls on the seawater intrusion wedge length and the nitrates concentration transmitted downstream from them were examined by Abo-Shaeshaa et al.^20^ utilising SEAWAT and MT3DMS.

Recent studies demonstrated that machine learning–based methods, combined with numerical modeling, are increasingly utilized to predict and manage seawater intrusion in coastal aquifers exaggerated by groundwater abstraction and subsurface engineered barriers. Overall, ensemble models (particularly gradient boosting methods) deliver highly accurate and interpretable predictions, reliably recognizing groundwater abstraction, recharge conditions, and subsurface barrier design parameters as the main controls on seawater intrusion dynamics^21–27^.

The mixing zone plays an important role in regulating the dynamics of subsurface flow and the water exchange between the groundwater and the ocean. Both the width of the mixing zone and the rate of mixing vary throughout the saltwater–freshwater interface^28^. Among the factors influencing the mixing zone are the mechanical dispersion, molecular diffusion, freshwater outflow, seawater influx, and density differential between the groundwater and the seawater^29^. Paster and Dagan^30^ claim that the steady state mixing zones are mostly caused by regional transverse dispersion. Oz et al.^31^ stated that the width of the saltwater–freshwater interface is determined by the transverse dispersivity. Badaruddin and Mehdizadeh^32^ found that an increase in the transverse dispersivity value led to a shear effect, which caused the mixing zone to move landward at the top and seaward at the bottom. Vertical fractures had little effect on the duration of the saltwater invasion; however, they had a considerable impact on the width of the mixing zone^33^. Eeman et al. (2011) suggest that longitudinal dispersion could have a greater effect on the mixing zone width than transverse dispersion. The relative contributions of each longitudinal and transverse dispersion to the freshwater-saltwater mixing zone are therefore not well understood. As noted by Narayanan and Eldho^34^, further investigation into the time-dependent formation of the mixing zone and its impact on convective saline circulation may be necessary. Abarca and Prabhakar Clement^35^, by calculating the geometrical average of the two dispersivity values, proposed that simultaneous longitudinal and transverse dispersion have a substantial impact on the mixing zone width Chang et al.^36^ used numerical simulation and laboratory tests to examine the dynamics of the seawater-freshwater mixing zone and conducted scenarios to compare between the mixing zone variations in cases of with and without subsurface physical barriers. The outcomes demonstrates that the construction of a subsurface barrier will not instantaneously slow down the velocity of saltwater intrusion and vary the distribution of salinity for the seawater-freshwater mixing zone. The block effect of cutoff with various bottom opening sizes scenarios became obvious only after the seawater intrusion wedge toe advanced to reach the cutoff wall.

However, previous studies on the impact of using underground dams in controlling seawater intrusion in coastal aquifers have neglected the dynamic behavior of seawater-freshwater mixing zone in sloping beach coastal aquifers. The main objective of this paper is to assess variations in the dynamics of the SWI mixing zone by conducting numerical simulations using the SEAWAT code, with particular attention paid to the variations of underground dam height and the angles of inclination of a sloping beach. The combined impact of the underground dam height, inclination angle of the beach face, hydraulic conductivity, longitudinal despersivity, and transverse despersivity were examined and includes the mixing zone (MZ) area, the bottom width of the mixing zone, the central width of the mixing zone, the average MZ width, the length of the 90% isohalines and the submarine groundwater discharge.

### Methodology

For modelling SWI in sloping beach coastal aquifers, the SEAWAT code^37^ was used in this work. Underground dams were used as a countermeasure to minimise SWI. The hydrodynamic behaviour of the freshwater–seawater mixing zone in sloping beach coastal aquifers was examined using the two-dimensional coastal aquifer of Chang et al.^36^. The model domain’s dimensions are 90 cm in length (x-direction), 27 cm in height (z-direction) and 5 cm in width (y-direction).

The model domain’s dimensions were discretised, with each cell measuring 0.5 cm by 0.5 cm. The freshwater boundary was allocated a constant value of 0.0 mg/l, whereas the saltwater boundary was assigned a constant concentration of 36,000 mg/l. Both the aquifer’s top and bottom were set to the scenario of no-flow boundaries. It was decided that the saltwater head should be 25.2 cm in the seawater boundary, and the freshwater head should be 26.1 cm in the freshwater boundary. The coastal aquifer’s hydraulic conductivity was determined to be 6 × 10^–3^ m/sec.

**Fig. 1:**
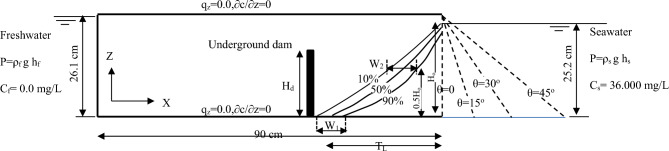
Schematic of the conceptual model. θ represents the inclination angle of the beach; H_d_ represents the depth of underground dam; T_L_ represents the saltwater wedge toe length. W_1_ and W_2_ represent the bottom and central width of the mixing zone, respectively.

The aquifer domain has a porosity of 0.4. The densities of freshwater and saltwater were determined to be 1000 and 1025 kg/m^3^, respectively. When dyed saltwater was tested, its viscosity was 0.001 kg/m.s. It was determined that the longitudinal dispersivity value was 0.15 cm, and the transversal dispersivity value was 10 percent of the longitudinal dispersivity^38^. A value of 1 × 10–9 m2/s was chosen for the molecular diffusion coefficient (D). Numerical stability is ensured due to fulfilment of the Peclet number condition (Pe = 3.3 < 4) on the part of the model domain cell and dispersivity. The height scenarios of the underground dam (H_d_ were 7 cm, 9 cm, 11 cm, 13cm and 15 cm, respectively. To investigate the impact of a sloping beach coastal aquifer, four varying values of beach inclination (θ were implemented; these were 0.0° (vertical boundary), 15°, 30° and 45°. The scenario of SWI without using the underground dam was also implemented to compare the effects of using underground dams on the hydrodynamic behaviour of SWI in sloping unconfined coastal aquifers. The simulation’s duration was set to be equal 180 min, and the time step of the simulation was set to be 1 min. Figure 1 shows the schematic diagram of the studied sloping beach coastal aquifer, and Table 1 shows the input hydrological parameters of the sloping beach coastal aquifer.

**Table1:** Parameter definitions.

Parameter	Definition
C_s_	Concentration of saltwater
C_f_	Concentration of freshwater
h_s_	Head of seawater
h_f_	Head of freshwater
ρ_s_	Density of seawater
ρ_f_	Density of freshwater
D	Molecular Diffusion coefficient
H_d_	Height of underground dam
θ	Inclination angle of sloping beach coastal aquifer
K	Hydraulic conductivity of the aquifer
H_a_	Saturated aquifer depth at the sea boundary
L_T_	Intruded length of the 50% isohaline
L_90%_	Length of the 90% normalised isohalines of SWI
W_1_	Width of the mixing zone at the bottom of the aquifer
W_2_	Width of the mixing zone at 50% of the saturated aquifer depth
MZW	Average width of the mixing zone
SGD	Subsurface groundwater discharge
A_M_	Area of the mixing zone
α_L_	Dispersivity in the longitudinal direction
α_T_	Dispersivity of the transverse direction

The domain dimensions were selected based on commonly used configurations in previous coastal aquifer modelling studies and are representative of typical hydrogeological settings^36^. Boundary heads were assigned to reproduce a realistic hydraulic gradient between inland recharge conditions and the coastal sea boundary^36^. The selected hydraulic conductivity value reflects average hydraulic behaviour commonly used in saltwater intrusion studies and is consistent with previously published modelling work^8,36^. The selected heights tested of the underground dam are 7, 9, 11, 13, and 15 cm with corresponding height ratio (H_d_/h_s_) equals 0.25, 0.35, 0.42, 0.5, and 0.58, respectively, consistent with the previously published papers^8,19,21,25–27,39^ The tested sloped angles 0.0°, 15°, 30°, and 45° were selected to represent typical geological conditions observed in coastal sedimentary aquifers and consistent with published research^11,13,40,41^.

### Evaluation indices

The proper management of coastal groundwater resources requires a detailed understanding of the mixing zone’s location and extent. The extent of the mixing zone in a sloping beach coastal aquifer has been assessed using seven distinct criteria under various beach inclinations and underground dam height scenarios. The SWI wedge toe’s length, denoted by the symbol T_L_, was determined by measuring the horizontal distance between the 50% isohaline (i.e., 50% of the seawater concentration in the seawater boundary) and the seawater boundary of the sloping beach coastal aquifer. This was done to determine the extent of SWI. Subsurface groundwater discharge (SGD) in a coastal aquifer is the flow of groundwater from the aquifer into the adjacent sea through the subsurface. The Zone Budget utility in SEAWAT was used to automatically compute the total discharge across selected boundary zones.

The length of the 90% normalised isohalines of the seawater concentration boundary is denoted by the notation L_90%_. The freshwater–seawater mixing zone can be assessed using the 10% and 90% normalised isohalines, per Abarca and Prabhakar Clement^35^ and Lu and Luo^42^. The region between the 10% and 90% isohalines of the saltwater concentration border is known as the mixing zone (A_M_). W_1_ and W_2_ represented the width of the freshwater–seawater mixing zone. The horizontal separation between the different isohalines at the base of the sloping beach coastal aquifer is known as the width of the mixing zone (W_1_). The horizontal distance between the different isohalines at the midpoint of the saturated depth of the sloping beach coastal aquifer is known as the width of the mixing zone (W_2_)^32^. According to Lu et al.^43^, the A_M_ divided by the L_90%_ (the length of the 90% isohaline) is the average width of the freshwater–seawater mixing zone (MZW), which is a metric used to assess the extent of the complete mixing zone.

### Mesh discretization and model verification

**Fig. 2:**
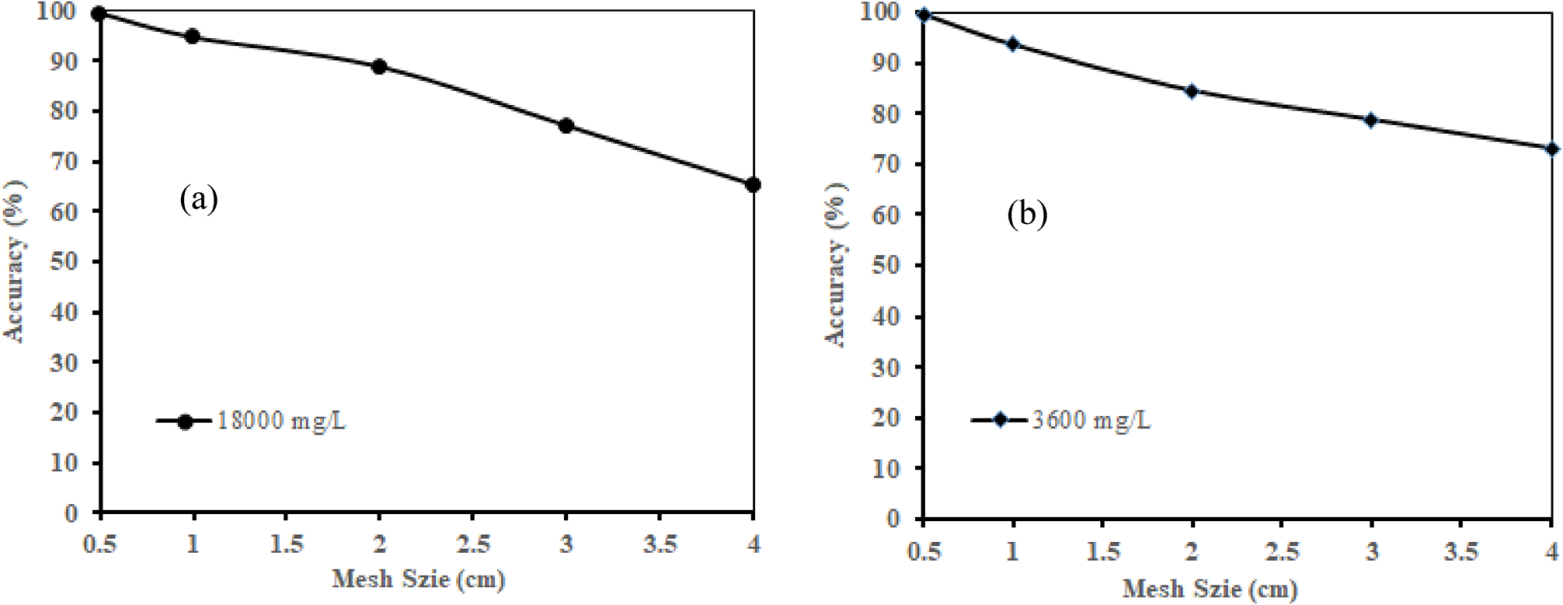
Accuracy of SEAWAT model results regarding to different assigned mesh size: (**a**) salt concentration contour line (18,000 mg/L), and (**b**) salt concentration contour line (3,6000 mg/L).

To check the grid SEAWAT sensitivity, the built model was run using SEAWAT code according to different grid sizes, involving 0.5 × 0.5, 1.0 × 1.0, 2.0 × 2.0, 3.0 × 3.0, and 4.0 × 4.0 cm. Also, the absolute error between the experimental saltwater intrusion wedge length^36^ for contour concentration line 18,000 mg/L and 3,600 mg/L and the simulated saltwater intrusion wedge length were calculated to check the SEAWAT model accuracy in respect to various meshing sizes. Figure 2 represents the calculated error for the length of saltwater intrusion wedge regarding different grid sizes for concentration 18,000 mg/L and 3,600 mg/L contour lines. From the figure results, it can be confirmed that reducing the grid model size enhances the SEAWAT model results accuracy. Additionally, for contour concentration line of 18,000 mg/L, the computed absolute error among the experimental and simulated results of saltwater intrusion wedge length equals 0.7, 5.4, 11.2, 22.9, and 34.5%, with achieving model accuracy equals to 99.3, 946, 88.8, 77.1, and 65.4% for grid sizes equals 0.5, 1, 2, 3, and 4 respectively. Also, for contour concentration of 3,600 mg/L, the calculated absolute error between the experimental and numerical results of saltwater intrusion wedge length equals 0.4, 6.4, 15.5, 21.2, and 26.9 by corresponding SEAWAT accuracy equals 99.6, 93.6, 84.4, 78.8, and 73.06 consequentially.

**Fig. 3:**
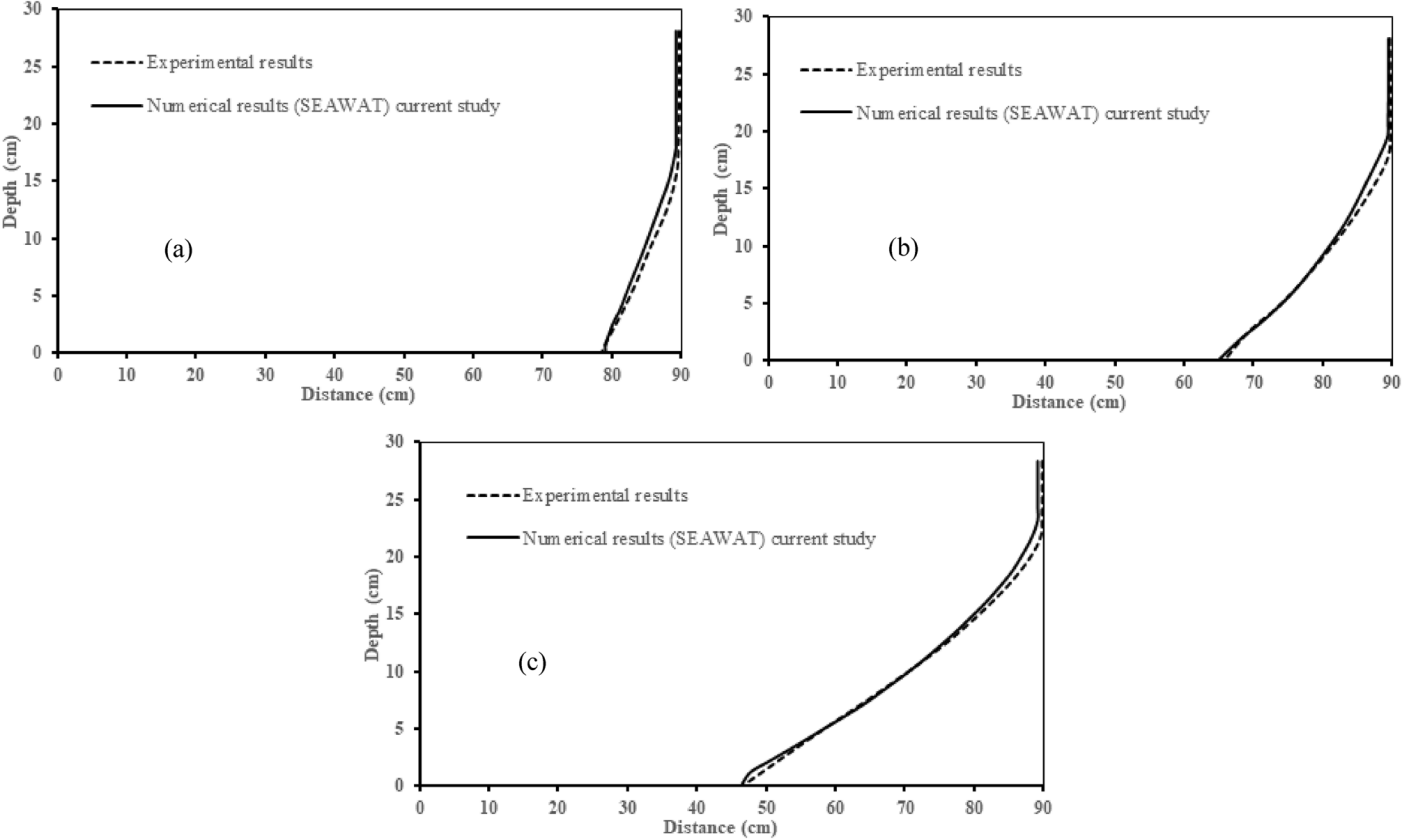
Comparison between simulated and experimental results of seawater-freshwater interface for salt concentration contour line (3,6000 mg/L) for model verification.

Figure 3 shows the comparison between the experimental results of seawater intrusion wedge Chang et al.^36^ and numerical results for the current study using SEAWAT code for model verification. The saltwater intrusion wedge for concentration line equals 18,000 mg/L were compared for various times were 5.0, 25, and 180 min to check the model accuracy for various time steps. For time step equals 5.0 min, the seawater intrusion wedge toe length at the bottom of the coastal aquifer equals 11.51 and 10.96 cm for numerical and experimental results, respectively with corresponding absolute error equals 0.05 cm. For time step equals 25 min, the seawater intrusion penetration length at the bottom of the coastal aquifer equals 65.8, and 65.04 cm for numerical and experimental results, respectively with corresponding absolute error equals 0.76 cm. In addition, at the end of the simulation at 180 min, the numerical and experimental penetration length of seawater intrusion wedge toe at the bottom of the aquifer equals 46.69, and 46.39 cm, respectively, with achieved absolute error equals 0.3 cm.

## Results and discussion

**Fig. 4:**
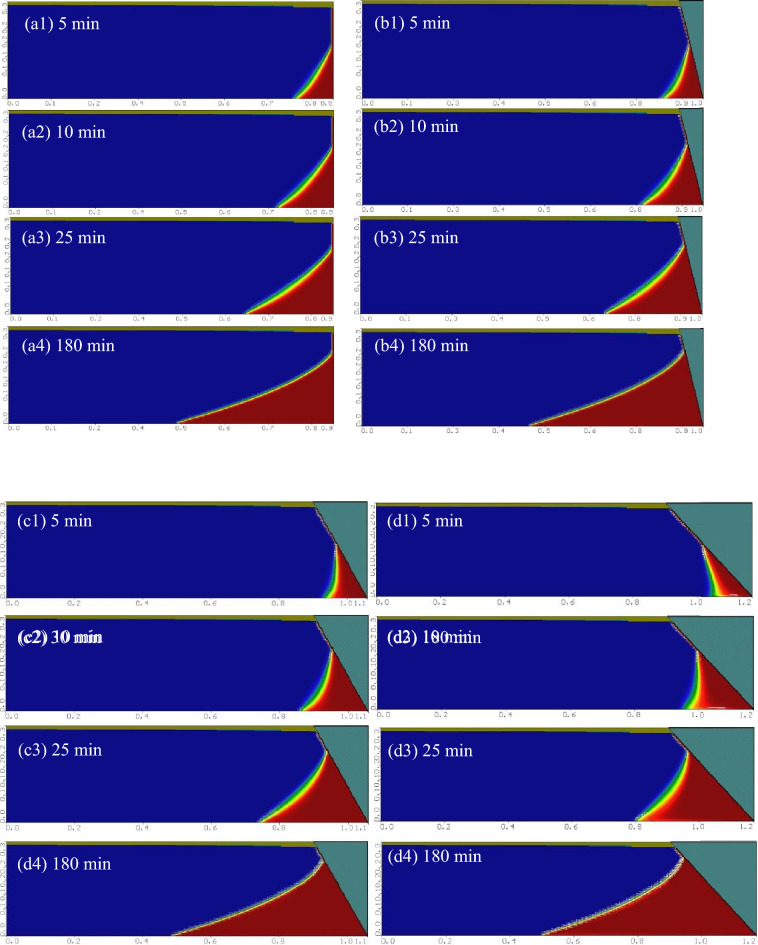
Steady state seawater intrusion for sloped beach coastal aquifers for the case of without an underground dam for various inclination angles of the beach face: (a) 0.0°, (b) 15°, (c) 30° and (d) 45°; the black lines indicate the 10, 50 and 90% isohalines of the mixing zone.

Figure 4 depicts the variation of the SWI wedge for sloped beach coastal aquifers for the case without an underground dam for various inclination angles of the beach coastal face of 0.0°, 15°, 30° and 45°. The black lines indicate the 10, 50 and 90% isohalines of the SWI mixing zone. In the case of vertical beach coastal aquifers (Fig. 2 a1, a2, and a3), the SWI process first begins at 5 min, when the width of the mixing zone is growing. Through the time up to 30 min, the SWI wedge moves further inland, and the width of the mixing zone increases. By the end of the simulation at 180 min, SWI reaches a steady state; the mixing zone has totally formed, and the width is decreased compared with the earlier intrusion time. With a sloping beach coastal aquifer (15°) (Fig. 2 b1, b2, and b3), SWI moves further inland, with a toe length higher than the case without an inclination, in the same distinct time (5, 30, and 180 min). The SWI mixing zone formed becomes wider compared with (0.0°) inclination. The SWI mixing zone formed becomes wider compared with (0.0°) inclination. The sloping geometry increases the shear effects along the saline wedge boundary, enhancing the dispersion and the dilution processes. The 45° slope shows the largest and most diffuse mixing zone. Isohalines are strongly elongated and widely separated, reflecting substantial transverse dispersion. Increasing the slope of the beach face to 30° (Fig. 2 c1, c2, and c3) and 45° (Fig. 2 d1, d2, and d3) inclination resulted in an increase in the length of the SWI wedge toe as well as the bottom mixing zone width (W_1_) and the central mixing zone width (W_2_) compared with the vertical boundary coastal aquifer (0.0°) as a result of the impact of inclination boundary in the SWI process.

**Fig. 5:**
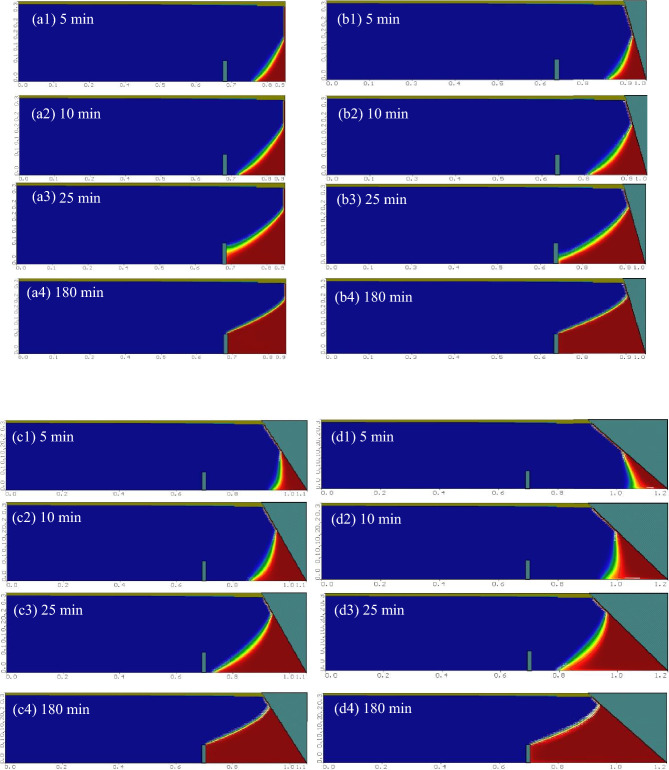
Steady state seawater intrusion for sloping beach coastal aquifers for underground dam height (H_d_) eqauls 7cm for various inclination angles of the beach face: (**a**) 0.0°, (**b**) 15°, (**c**) 30° and (**d**) 45°; the black lines indicate the 10, 50 and 90% isohalines of the mixing zone.

Figure 5 shows the advancement of the SWI wedge for slope beach coastal aquifers with an underground dam height (H_d_) = 7 cm, for various inclination angles of the beach coastal face of 0.0°, 15°, 30°, = and 45°; the black lines indicate the 10, 50 and 90% isohalines of the mixing zone.

With embedment of an underground dam with a height equal to 7 cm and with inclination angle (0.0°) (Fig. 5 a1, a2 and a3), the SWI process starts in the first 5 min, and the width of the mixing zone grows. In the earlier time of the simulation, no impact of the underground dam on the SWI mixing zone is noticeable. The block impact of the underground dam became noticeable only after the SWI wedge toe advances to the location of the underground dam. It can be shown that the area of the mixing zone increases with time; nevertheless, it afterward declined at the approach of the SWI toe to the location of the underground dam.

Increasing the inclination angle of the sloping beach face from 0.0° to 15° (Fig. 5 b1, b2 and b3) led to a noticeable delay of SWI due to the impact of the inclination boundary. Initially, the SWI wedge intruded into the aquifer over time; the impact of the inclination angle is obvious, where SWI advances further inland into the aquifer. In addition, up to 30 min after the SWI wedge toe is delivered to the underground dam location, the impact of the barrier is noticeable, where the SWI toe takes a longer time to reach the dam, and the height of SWI in the dam location is lower than the case of a 0.0° inclination angle. The mixing zone area in the case of an inclination becomes wider with SWI at inclination angle of 0.0°, and finally at 180 min, the SWI wedge reaches a steady state with the mixing zone width nearest to the case of 0.0° inclination.

Also, for a sloping beach face of 30^o^ (Fig. 5 c1, c2 and c3) and 45^o^ (Fig. 5 d1, d2 and d3) no impact of subsurface barrier on the SWI intrusion is noticeable at the beginning of the simulation. The inclination angle affects the velocity of the intrusion, SWI moves faster compared with a beach of lower inclination angles. Increasing the inclination angle of the sloping beach face to 30° and 45° resulted in an increase in the area and the width of the mixing zone in the earlier part of the simulation, in comparison with the lower slope angles. The block impact of the underground dam was delayed, where the wedge toe just reached the dam location at 30 min with the inclination angle 30°, and the SWI wedge toe takes longer than 30 min, with a longer toe length, in the case of a 45° inclination. As a result, the 45° inclination achieved a delayed response to an underground dam block compared with cases with lower inclination angles. The inclination of the sloping beach face to 45° leads to a noticeably wider mixing zone and a larger area mixing zone in early time of intrusion, but at the end it reached a steady state with similar conditions as the mixing zone with lower sloping angles.

**Fig. 6:**
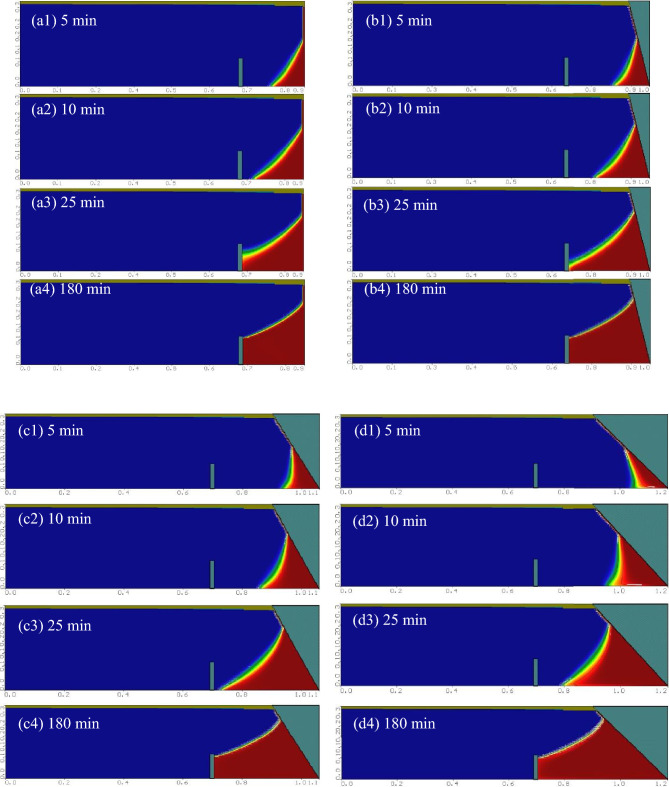
Steady state seawater intrusion for slope beach coastal aquifers for an underground dam height of (H_d_) = 9cm for various inclination angles of beach face: (**a**) 0.0°, (**b**) 15°, (**c**) 30° and (**d**) 45°; the black lines indicate the 10, 50 and 90% isohalines of the mixing zone.

Figure 6 displays the progression of the SWI wedge for slope beach coastal aquifers for underground dam height of (H_d_) = 9 cm, for various inclination angles of the beach coastal face at 0.0°, 15°, 30° and 45°; the black lines indicate the 10, 50 and 90% isohalines of the mixing zone. Early in the simulation, at 5 min, the impact of the underground dam height on the SWI mixing zone at different sloping angles is not noticeable. The presence of the underground dam evidently disturbed the flow in the area around its location. Nevertheless, the fields of flow far from the location of the underground dam displayed comparable traits in several instances of underground dam heights. As a result, the simulated SWI wedge toe early on was found to be similar in various underground dam scenarios.

The block impact of SWI started when the wedge toe reached the actual underground dam. Comparing the results in Fig. 3 for dam height H_d_ = 7 cm with Fig. 6 for dam height H_d_ = 9 cm confirmed that when the SWI wedge toe reached the dam, the mixing zone formed began to rise more quickly with increasing the height of the dam. The elevation of the mixing zone with dam height H_d_ = 9 cm is higher than with H_d_ = 7 cm. By 180 min, at the end of the simulation, the mixing zone reached a steady state with the highest elevation compared with the earlier time of the simulation. In addition, increasing the inclination angle of the sloping beach face to 15°, 30° and 45° resulted in an increase in the length of L_90%_ over time because of the shape of the inclination seawater boundary compared with the vertical boundary.

**Fig. 7:**
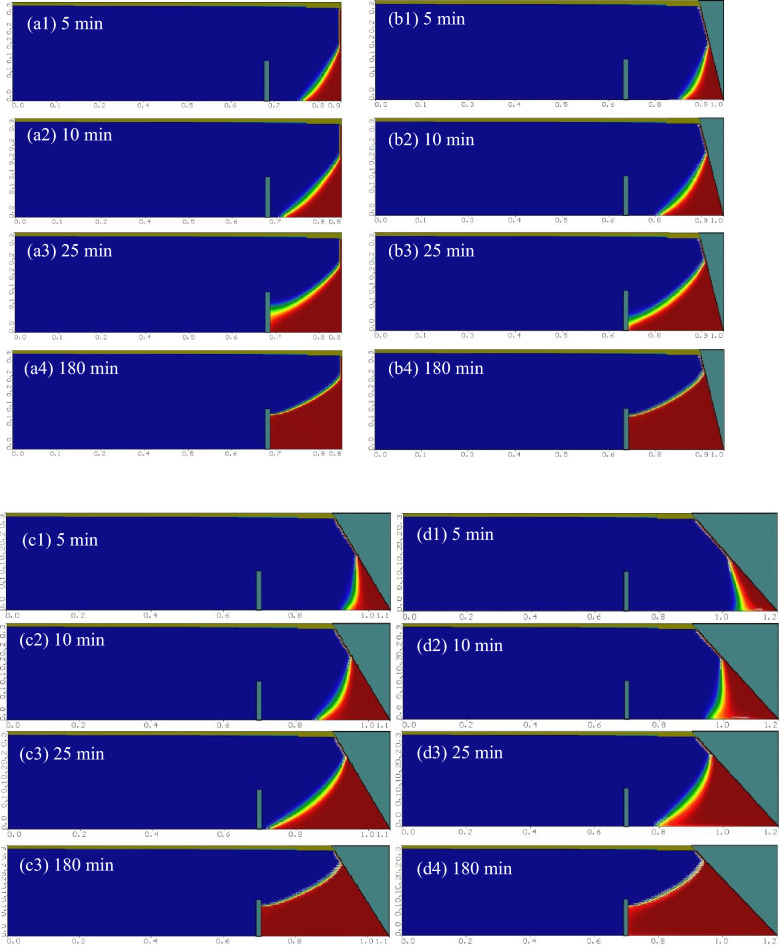
Steady state seawater intrusion for slope beach coastal aquifers for an underground dam height of (H_d_) = 11cm for various inclination angles of beach face: (**a**) 0.0°, (**b**) 15°, (**c**) 30° and (**d**) 45°; the black lines indicate the 10, 50 and 90% isohalines of the mixing zone.

Figure 7 illustrates the progress of the SWI wedge for slope beach coastal aquifers for underground dam height (H_d_) = 11 cm, for different scenarios of inclination angle of the beach coastal face of 0.0°, 15°, 30° and 45°; the black lines indicate the 10, 50 and 90% isohalines of the mixing zone. Increasing the height of the underground dam to 11 cm (Fig. 7) has a noticeable impact on the shape of the SWI mixing zone formed. In the initial stage of simulation, no significant variation in the seawater intrusion wedge is detected compared with no-dam scenario. In addition, when the seawater intrusion wedge arrived the bottom of the underground dam, the block impacts of the underground dam gradually observed. Also, the seawater-freshwater mixing zone displayed similarities regardless of the presence of an underground dam during the initial stage of simulation. After the arrival of seawater intrusion wedge toe at the underground dam location, the seawater-freshwater mixing zone firstly expanded because of the slow of groundwater flow close to the underground dam. Comparing the results of Figs. 5, 6 and 7 for the same inclination angle of the sloping beach confirmed that the length of the SWI wedge T_L_ remained constant throughout the intrusion process, which suggests that the impact of the height of the underground dam on the SWI velocity was not easily observable.

Increasing the underground dam height H_d_ to 11 cm resulted in an increase in the steady state elevation of the SWI wedge at the location of the underground dam compared with H_d_ = 7 and 9 cm. For all scenarios of the sloping beach face inclination angle, the bottom width of the SWI mixing zone at the end of simulation exhibited a constant value when the SWI wedge toe reached the dam location as a result of the impact of the dam block.

**Fig. 8:**
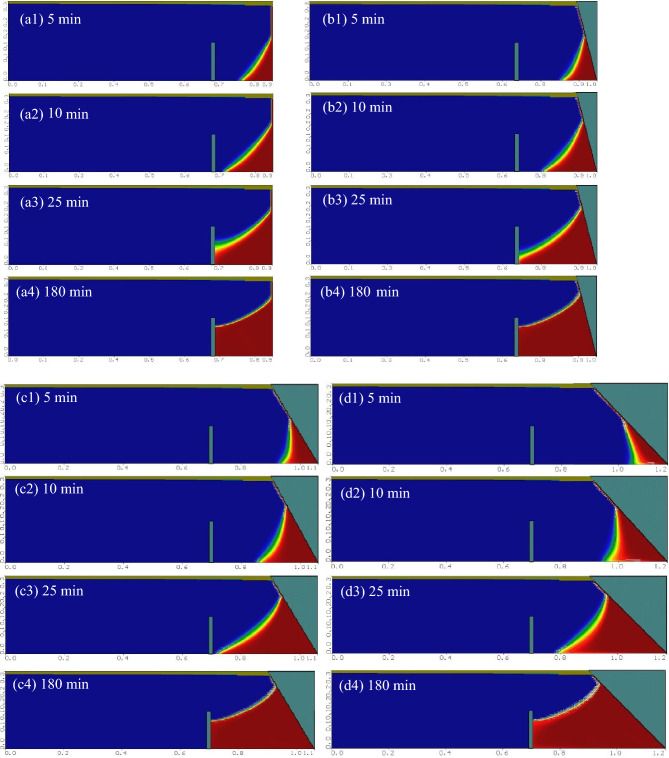
Steady state seawater intrusion for slope beach coastal aquifers for an underground dam height of (H_d_) = 13cm for various inclination angles of the beach face: (**a**) 0.0°, (**b**) 15°, (**c**) 30° and (**d**) 45°; the black lines indicate the 10, 50 and 90% isohalines of the mixing zone.

**Fig. 9:**
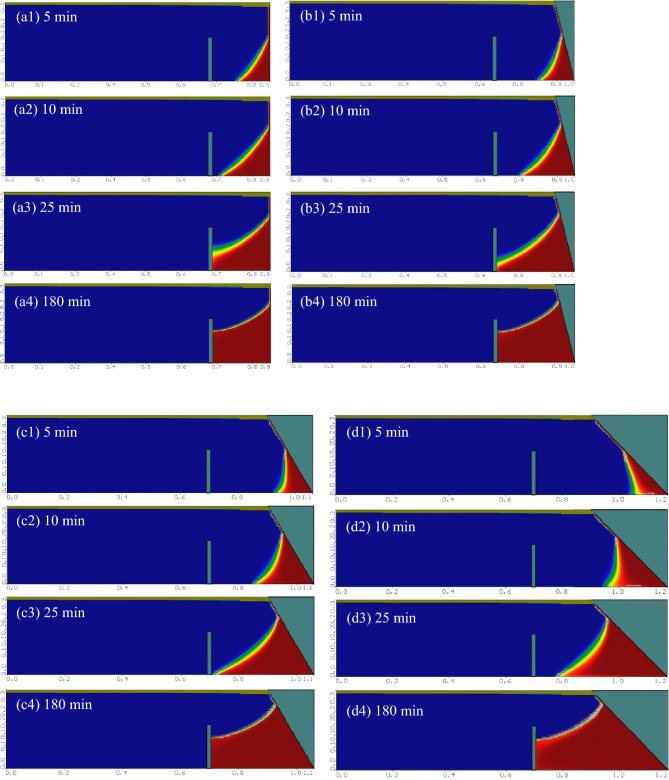
Steady state seawater intrusion for slope beach coastal aquifers for an underground dam height of (H_d_) = 15cm for various inclination angles of the beach face: (**a**) 0.0°, (**b**) 15°, (**c**) 30° and (**d**) 45°; the black lines indicate the 10, 50 and 90% isohalines of the mixing zone.

Figure 8 and Fig. 9 display the advancement of SWI for slope beach coastal aquifers for an underground dam height of (H_d_) = 13cm and15 cm, respectively, for various inclination angles of the beach face equal to 0.0°, 15°, 30° and 45°; the black lines indicate the 10, 50 and 90% isohalines of the mixing zone. The steady state bottom width of the mixing zone (W_1_) and the central width (W_2_) at the end of simulation were constant for various inclination angles of the sloping beach. The impact of the height of the underground dam is obvious on the delayed time; where the seawater wedge reached the underground dam location, the final elevation of the SWI wedge increased with embedment of the underground dam with higher heights. The maximum elevation of the SWI wedge toe at the dam location was observed with a dam height H_d_ equal to 15 cm. No changes to the SWI wedge height was observed at the dam location when varying the inclination angle of the sloping beach. On the other hand, the elevation of the SWI wedge increased at the centre of the saturated depth of the aquifer upon increasing the inclination angle of the sloping beach for the same constant case of underground dam height.

**Fig. 10:**
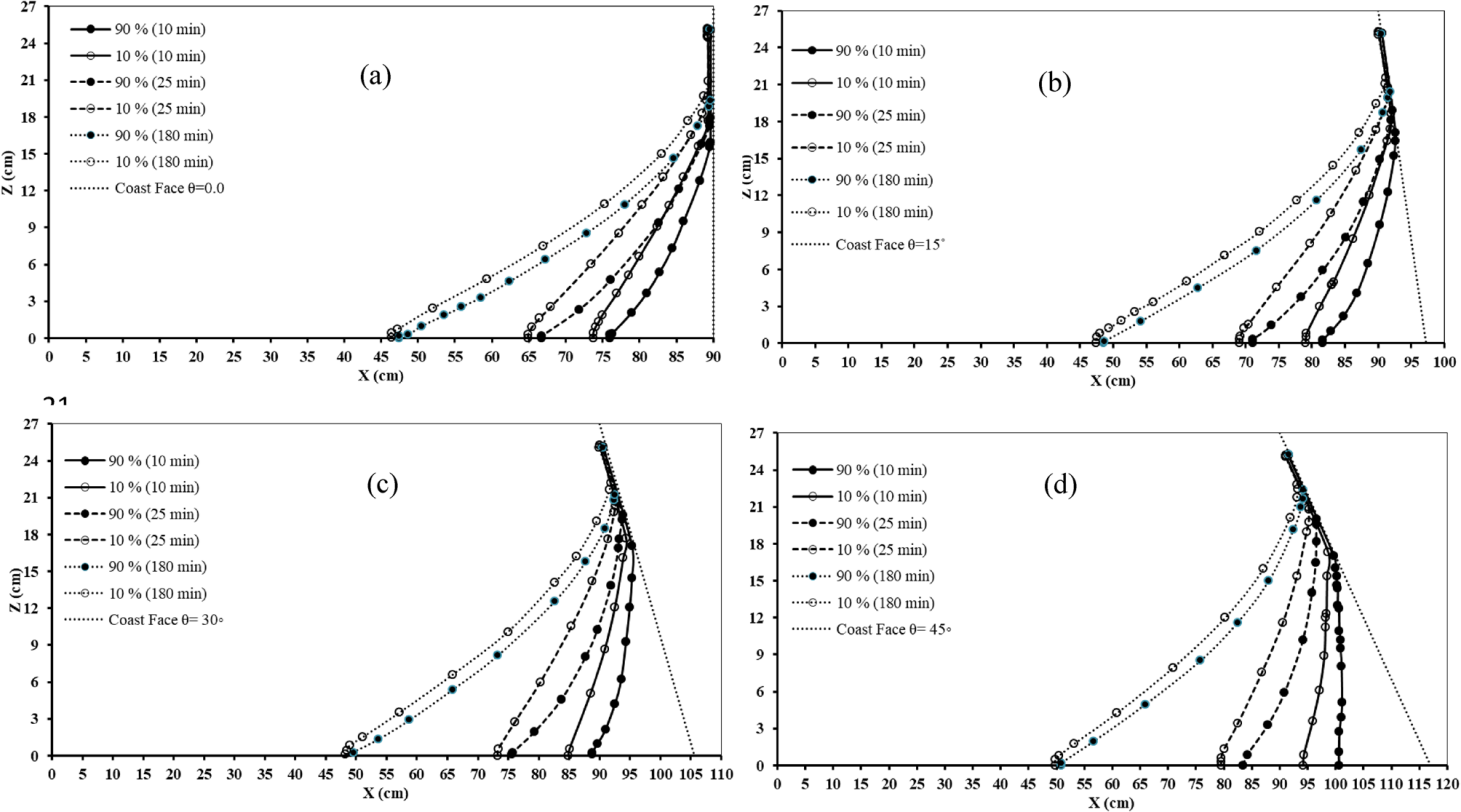
Hydrodynamic behaviour of the seawater–freshwater mixing zone for different inclination angles of the beach: (**a**) 0.0°, (**b**) 15°, (**c**) 30° and (**d**) 45°.

Figure 10 displays the progress of the seawater–freshwater mixing zone over time for the case of SWI without using an underground dam for four various coastal beach slope angles: 0°, 15°, 30° and 45°. The progression of isohalines that represent salinity levels of 10% and 90% at various time intervals (10, 25 and 180 min) is depicted in each subfigure (a–d), indicating one inclination angle. The seawater–freshwater mixing zone is described by the region incorporated between the 10% and 90% isohalines, which also acts as a stand-in for evaluating SWI dynamics. In every instance, the mixing zone grows landward over time, representing the slow inland advancement of saltwater brought on by boundary conditions and density-driven flow. The mixing zone is comparatively constrained in both the vertical and horizontal extents at a beach slope aquifer of 0° (Fig. 10a), suggesting limited penetration of inland saltwater. A wider mixing region is produced by raising the slope to 15° (Fig. 10b), which suggests improved lateral dispersion and a longer interface resulting from higher hydraulic gradients and changed flow routes. At 30° and 45° inclinations (Fig. 10c and d, respectively), where the isohalines become steeper and more dispersed, this pattern becomes more noticeable. The mixing zone size is significantly increased, especially around 45°, suggesting that steeper slopes promote more mixing and seawater advances inland.

**Fig. 11:**
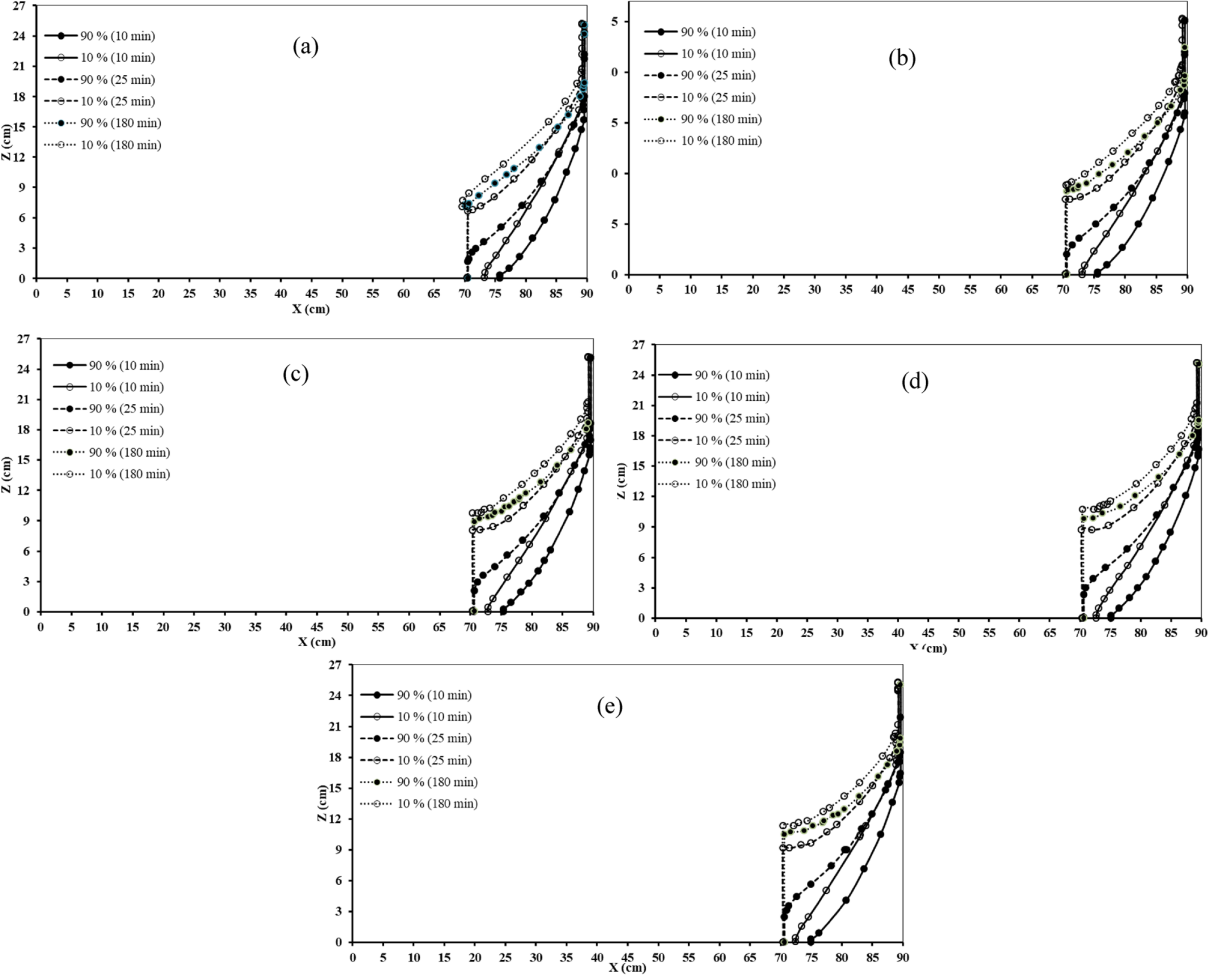
Hydrodynamic behaviour of the seawater–freshwater mixing zone for an inclination angle of the beach equal to 0.0°, for different underground dam heights (H_d_): (**a**) 7 cm, (**b**) 9 cm, (**c**) 11 cm, (**d**) 13 cm and (**e**) 15 cm.

Figure 11 shows the temporal evaluation of the seawater–freshwater mixing zone over time for different underground dam heights (H_d_), with a vertical beach slope coastal aquifer (0°). The advancement of isohalines that represent salinity levels of 10% and 90% at various time intervals (10, 25 and 180 min) is depicted in each subfigure (a–d), indicating one height of the underground dam. The seawater–freshwater mixing zone is described by the region incorporated between the 10% and 90% isohalines, which also acts as a stand-in for evaluating SWI dynamics. From the results of Fig. 11; the underground dam does not have any obvious impact on the seawater–freshwater mixing zone in the earlier period of the simulation. In addition, the impact became obvious when the toe of the SWI mixing zone reached the underground dam; after that a noticeable rise in the mixing zone occurred. Increasing the height of the underground dam resulted in an increase in the elevation of the mixing zone in the end of simulation. The bottom width of the mixing zone increased with the passage of time and minimised because of the accumulation of the seawater water upstream from the dam. On the other hand, the central width of the mixing zone became wider but was minimised when the toe of the SWI mixing zone reached the top of the dam and achieved a steady state.

**Fig. 12:**
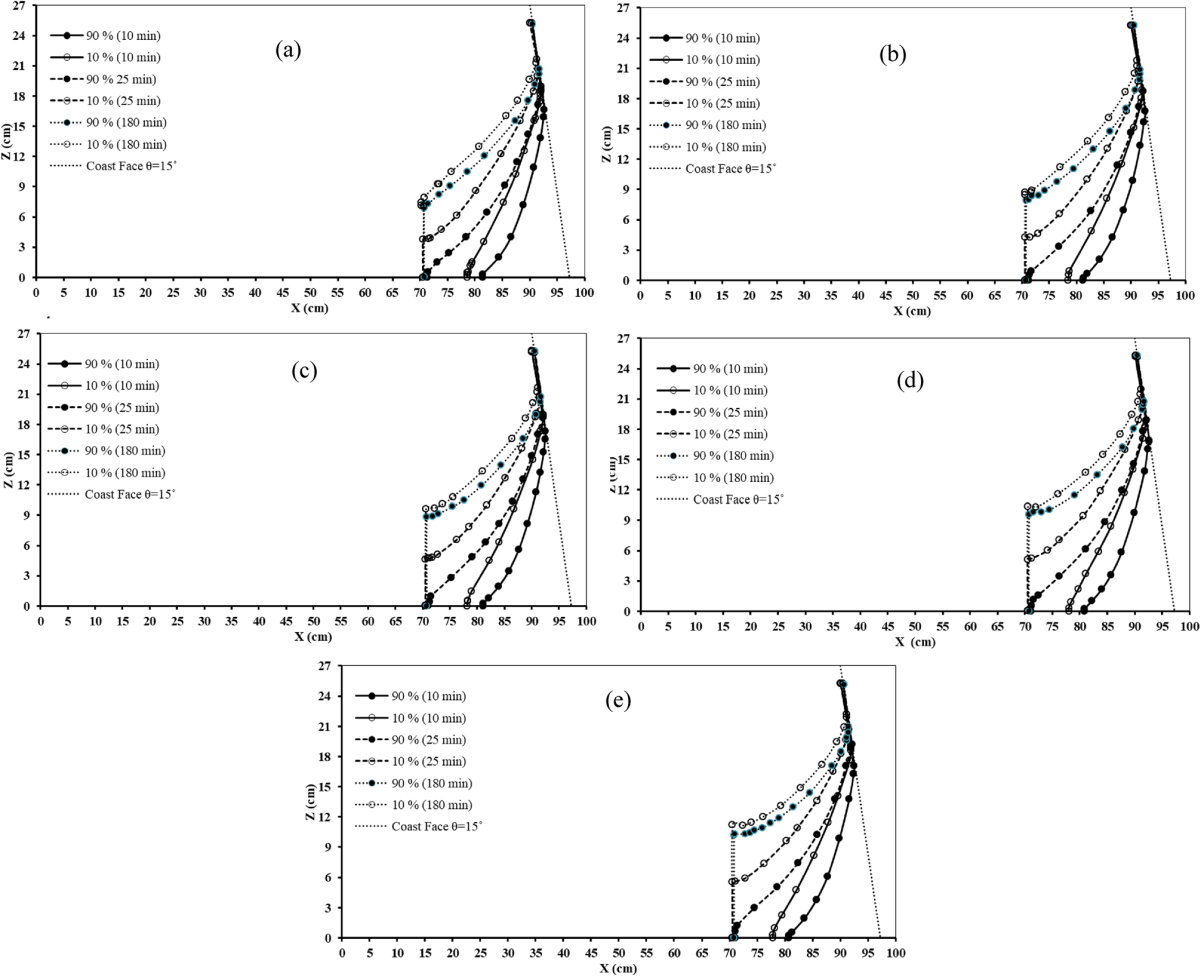
Hydrodynamic behaviour of seawater–freshwater mixing zone for an inclination angle of the beach equal to 15° for different underground dam heights (H_d_): (**a**) 7 cm, (**b**) 9 cm, (**c**) 11 cm, (**d**) 13 cm and (**e**) 15 cm.

The seawater–freshwater mixing zone’s development over time across varying depths of an underground dam (H_d_) in a coastal aquifer with a 15° slope is depicted in Fig. 12. Increasing the inclination angle of the beach face from 0.0° to 15° resulted in slowing the advancement of SWI inland into the coastal aquifer. The toe of the SWI mixing zone takes a longer time to reach the bottom of the underground dam compared with the vertical beach coastal aquifer. The length of the 90% isohaline of the SWI mixing zone obviously increased with an inclination of 15°, as a result of the inclination of the beach face boundary. No impact on the mixing zone is evident for an underground dam in the early part of the simulation. In addition, the effect of the underground dam becomes obvious when the SWI toe advanced to the dam location. At this point, the accumulation of the seawater behind the dam, as well as the elevation of the SWI mixing zone, rise with time until they reach the steady state. At the end of the simulation, the bottom width of the mixing zone is similar for various heights of the underground dam. In addition, a slight increase occurs in the central width of the mixing zone with increasing height of the underground dam. In respect to the area of the mixing zone, the increase resulting from the beach face inclination of the seawater boundary is pronounced. The maximum elevation of the SWI mixing zone is observed for the highest underground dam of H_d_ = 15 cm compared with the other cases for the same inclination angle case. For the same depth of the underground dam, increasing the inclination angle from 0.0° to 15° led to an increase in the elevation of the SWI mixing zone the middle distance between the underground dam and the seawater boundary.

**Fig. 13:**
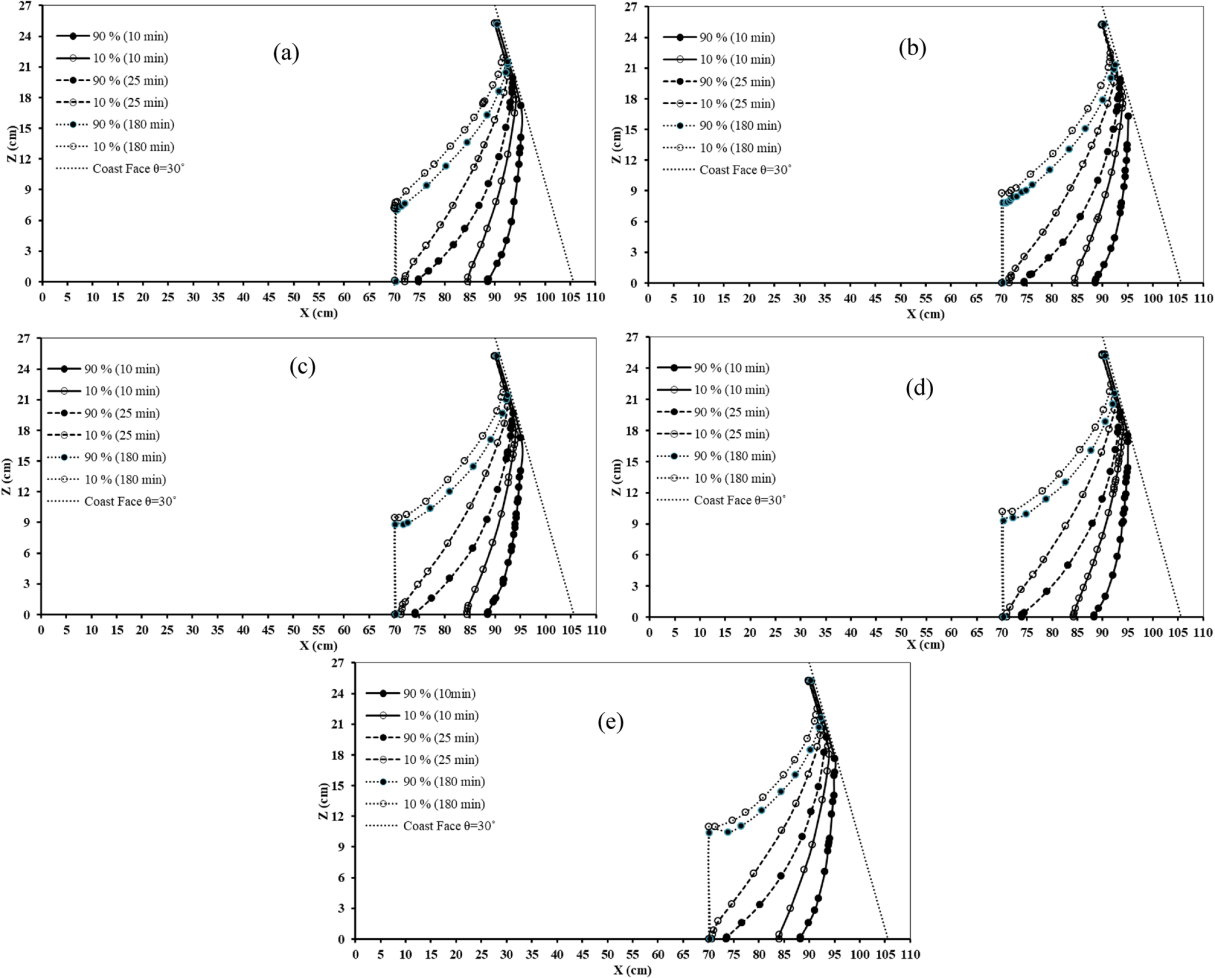
Hydrodynamic behaviour of the seawater–freshwater mixing zone for the inclination angle of the beach equal to 30° for different underground dam heights (H_d_): (**a**) 7 cm, (**b**) 9 cm, (**c**) 11 cm, (**d**) 13 cm and (**e**) 15 cm.

Figure 13 displays the temporal evaluation of the seawater–freshwater mixing zone over time for various underground dam heights (H_d_), with a coastal aquifer with an inclination angle of 30°. The seawater–freshwater mixing zone is described by the region incorporated between the 10% and 90% isohalines, which also acts as a stand-in for evaluating SWI dynamics and presents at various time intervals (10, 25 and 180 min). The impact of the underground dam block to SWI begins after 25 min from the start of simulation for an inclination angle of 30° compared with 15 min and 12 min for 15° and 0.0° inclination, respectively. Increasing the sloping beach face to 30° led to obvious increase in the initial bottom width and central width of the SWI mixing zone early in the simulation. However, there is no obvious impact of increasing the dam height on the mixing zone width for the same inclination angle of the beach coastal aquifer case. The length of the 90% isohaline of the SWI mixing zone enlarged upon varying the beach face from 0.0° to 30°. The elevation of the SWI mixing zone in the middle distance between the dam and the seawater boundary increased because of the beach face inclination. The steady state values of the central mixing zone width are close to being similar for various inclination beach faces.

**Fig. 14:**
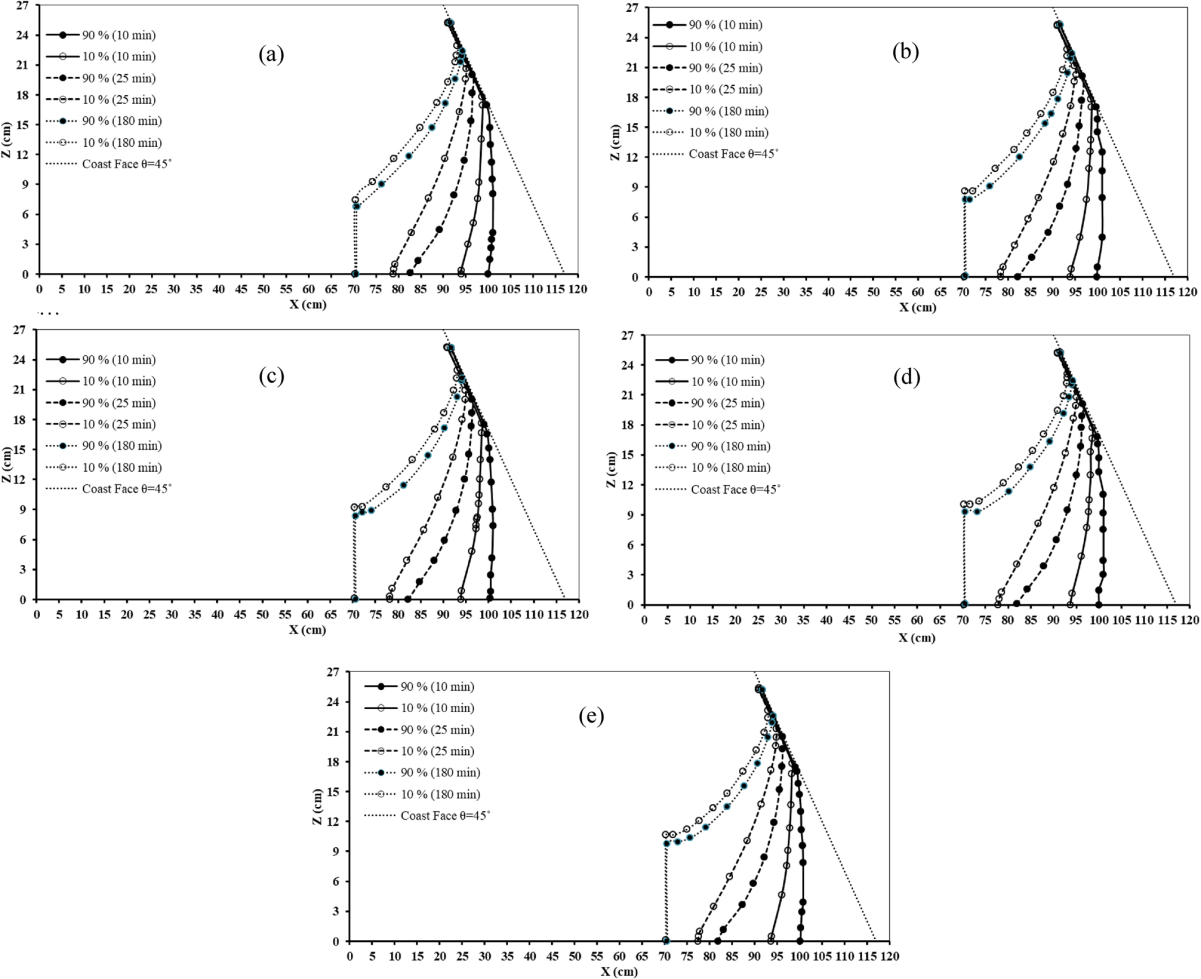
Hydrodynamic behaviour of the seawater–freshwater mixing zone for the inclination angle of the beach equal to 45° for different underground dam heights (H_d_): (**a**) 7 cm, (**b**) 9 cm, (**c**) 11 cm, (**d**) 13 cm and (**e**) 15 cm.

Figure 14 shows the progress of the seawater–freshwater mixing zone over time for five different underground dam heights (H_d_) for a coastal aquifer with an inclination beach angle of 45°. The seawater–freshwater mixing zone is described by the region incorporated between the 10% and 90% isohalines, which also acts as a stand-in for assessing the SWI dynamics and is depicted at various time intervals (10, 25 and 180 min). The area of the SWI wedge enlarges upon increasing the inclination angle of the beach face of coastal aquifer to 45 ^o^. Increasing the angle to 45° resulted in a delay of the progress of the SWI process inland into the aquifer because of the inclination angle. The seawater intrudes into the aquifer at a slower rate compared with vertical beach aquifer. As a result, the block impact of the underground dam started with a delay (after 30 min) compared with previous inclination angle cases of 0.0°, 15° and 30°. The underground dam does not have any obvious impact on the SWI intrusion process in the earlier stages of the simulation. Thus, the shape of mixing zone, bottom width and central width of the mixing zone exhibited similar characteristics for various heights of the underground dam at the same inclination angle of the beach face. This case of inclination achieved the maximum area of SWI, length of the 90% isohaline and the bottom width of the SWI mixing zone and the central width of mixing zone in comparison with the beach face inclination angles of 0.0°, 15° and 30°. The increase of the height of underground dam from 7 to 15 cm led to an increase in the elevation of the SWI mixing zone at the dam location. In addition, the maximum elevation was observed for H_d_ = 15 cm and inclination angle 45° from all tested scenarios. The existence of the underground dam and the obstruction effect also caused the SWI mixing zone to accumulate more slowly with higher inclination angle faces compared with the vertical boundary coastal face.

### Hydrodynamic behaviour of subsurface groundwater discharge in sloping beach coastal aquifers

**Fig. 15:**
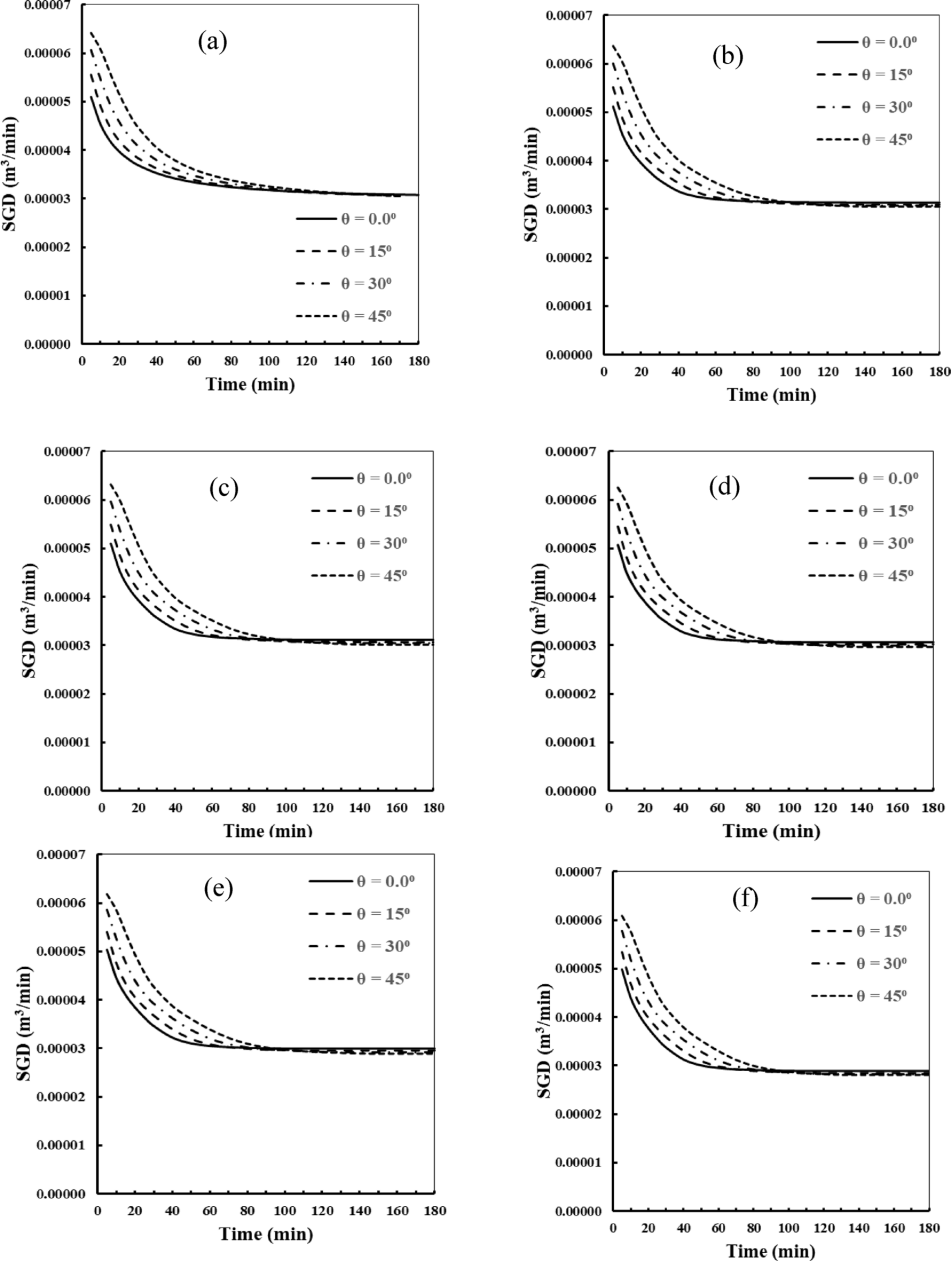
Hydrodynamic behaviour of subsurface groundwater discharge (SGD) for different values of sloping beach coastal aquifers θ = 0.0°, 15°, 30° and 45° for different cases of underground dam height (H_d_): (**a**) 0.0 cm, (**b**) 7 cm, (**c**) 9 cm, (**d**)11 cm, (**e**) 13 cm and (**f**) 15 cm.

Figure 15 displays the hydrodynamic behaviour of subsurface groundwater discharge (SGD) for different values of sloping beach coastal aquifers (θ) – 0.0°, 15°, 30° and 45° – for different cases of underground dam height (H_d_) at 0.0 cm, 7 cm, 9 cm, 11 cm, 13 cm and 15 cm. The results explain how the embedment of an underground dam slightly reduced the subsurface groundwater discharge compared with the case without a dam. Generally, the values of SGD decreased gradually in the initial stage for different dam height scenarios, particularly after the SWI wedge toe extended to the underground dam location. For H_d_ = 7 cm and θ = 0°, the SGD values declined gradually from 0.00005 m^3^/min at the beginning of simulation to reach 0.000033 m^3^/min at 40 min. For H_d_ = 7 cm, and θ = 45°, the SGD values declined gradually from 0.000065 m^3^/min at the beginning of simulation to reach 0.000033 m^3^/min at 40 min. In addition, after the accumulation of the seawater behind the dam, the SWI mixing zone rose as an effect of the dam block, and the SGD values gradually converged to a steady state condition. Increasing the dam height resulted in a slight reduction of the subsurface groundwater discharge. The underground dam had little impact on the variation of the subsurface groundwater discharge. After 100 min from the start, the SGD reached a steady state at a lower rate in the highest dams compared with lowest dams. Subsequently, the SGD in the scenarios with a higher underground dam height showed a more rapid drop and finally reached a lower flow level. The SGD values also declined gradually for various scenarios of beach faces angles. In addition, increasing the angle of inclination of the sloping beach led to a slower reduction of the SGD rates than with a vertical beach boundary face. The SGD values reached a steady state later upon increasing the beach inclination angle more than with the vertical boundary beach. This is because the inclination angle in the boundary slowed the SWI process, meaning the seawater takes a longer time to reach the underground dam. In addition, the dam block effect starts to become obvious after the SWI toe reached the dam.

Figure 15 displays that the SGD declined progressively with increasing time for all inclination beach angles, with the most obvious reduction occurring within the first 40–60 min. At the early stage, the SGD at θ = 45° was around 20–35% higher than that at θ = 0.0°, however the variation among different angles became negligible after nearly 100 min as the curves converged toward a steady-state value of about 0.00003 m^3^/min. For Fig. 15.a, without an underground dam, the SGD gradually declined after 120 min and approached a steady state after a significant initial decrease under all slope conditions. An increase of 62.5% between the lowest and highest slopes is indicated by the initial SGD (at time = 0 min), which rose from nearly 0.00004 m^3^/min at θ = 0° to nearly 0.000065 m^3^/min at θ = 45° as the slope angle increase. The rate of loss was most pronounced in the first 60 min, and SGD declined more rapidly on steeper slopes early in the simulation. By the end of the simulation (180 min), the SGD values on all slopes converged to a narrow range of roughly 0.000033 m^3^/min, indicating that the differences between the different slope scenarios are less than 10%. In addition, for cases with an underground dam (Fig. 15b to f) the SGD showed the same trends, but the presence of an underground dam had little impact on the final values of SGD upon reaching a steady state. The values of SGD for various underground dam heights (H_d_) reached the steady state earlier when compared with the no-dam case and the SGD value was a little lower than without an underground dam for various inclination angles of sloped beach.

### Hydrodynamic behaviour of the seawater intrusion wedge length (50%) in sloping beach coastal aquifers

**Fig. 16:**
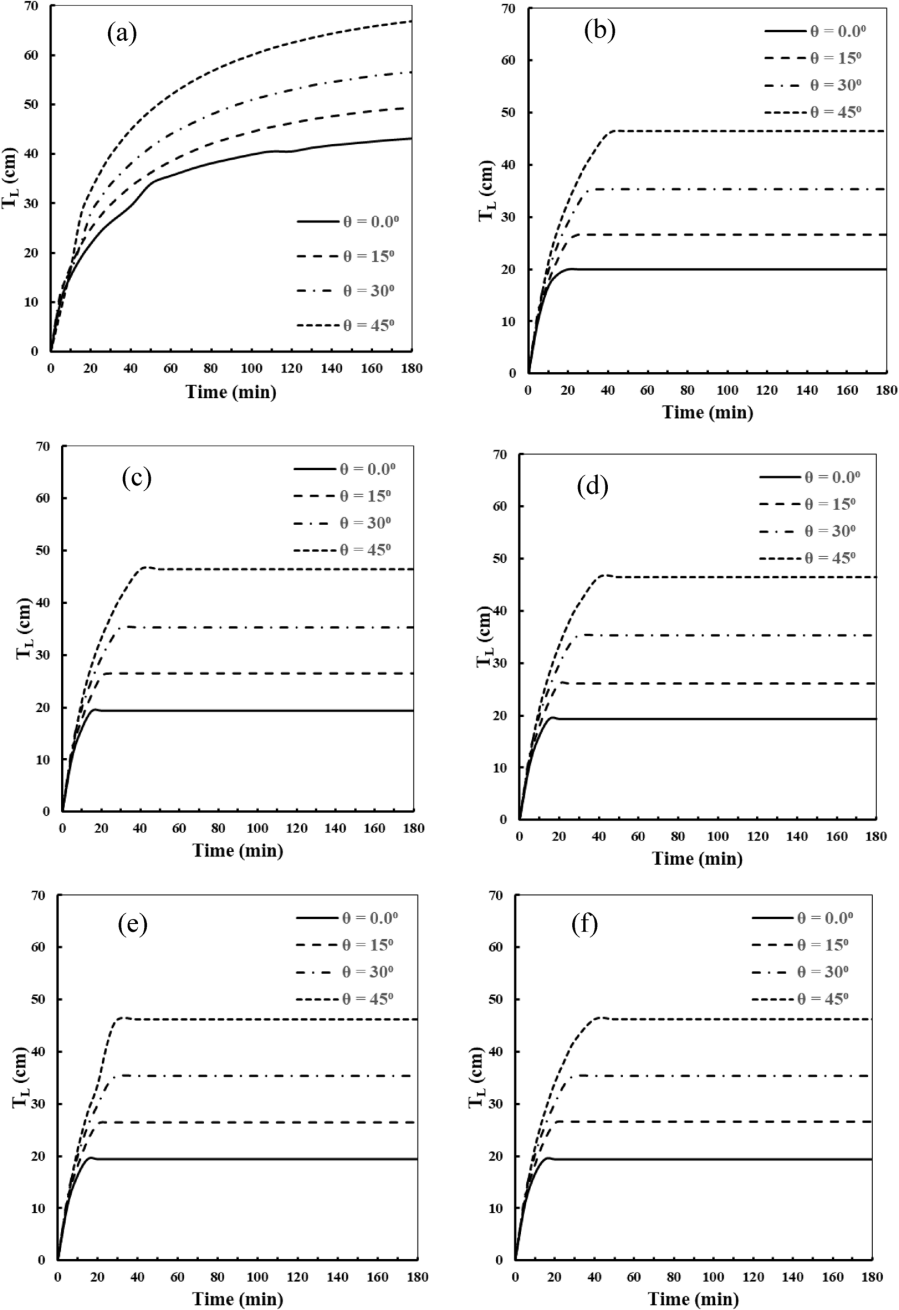
Hydrodynamic behaviour of seawater intrusion wedge length (50%) for different values of sloping beach coastal aquifers: θ = 0.0°, 15°, 30° and 45°, for different cases of underground dam height (H_d_): (**a**) 0.0 cm, (**b**) 7 cm, (**c**) 9 cm, (**d**)11 cm, (**e**) 13 cm and (**f**) 15 cm.

Figure 16 displays the hydrodynamic behaviour of the seawater intrusion wedge length (50%) for different values of sloping beach coastal aquifers (θ) at 0.0°, 15°, 30° and 45° for various cases of an underground dam height (H_d_) of 0.0 cm, 7 cm, 9 cm, 11 cm, 13 cm and 15 cm. For Fig. 7.a, with no underground dam, the T_L_ value increased over time with the advancement of SWI inland into the aquifer domain. There was no obstruction for the movement of the seawater, so the length increased gradually until it reached a steady state. Increasing the inclination angle of the beach face slows the intrusion process compared with the vertical boundary case. No impact was observed for the inclination on the T_L_ value in the initial time of simulation. In addition, increasing the angle of inclination from 0.0° to 45° resulted in an increase in the length of the 50% isohalines. For cases with an underground dam (Fig. 9.b to f), in the initial stage of the simulation the length of the 50% isohalines increased over the time due to the SWI process. After the SWI wedge toe reached the dam location, the dam blocked the advancement of the SWI toe inland into the aquifer domain, and the intrusion length reached a steady state. With an increasing angle of the beach face, the time that the SWI toe takes to reach a steady state increased. This is because the inclination angle of the seawater boundary caused an increase in the distance from the boundary to the underground dam. In addition, the impact of increasing the height of the underground dam on the T_L_ value is less significant. For various dam height scenarios, the T_L_ values reached a steady state under quite similar condition for the same value of the inclination angle.

In each instance for the inclination angle, in Fig. 16.a without subsurface barrier, T_L_ initially increased rapidly before progressively approaching a quasi-steady state. The initial pattern was the same for all slope conditions, but T_L_ moved significantly more quickly for steeper slopes (θ = 45°). In comparison to the θ = 0° slope condition, T_L_ increased by over 63% at the end of the 60-min period, reaching roughly 38 cm for θ = 0° and 62 cm for θ = 45°. This implies, particularly in the early transitory phases, that there is a positive correlation between the beach slope angle and the extent of SWI. As T_L_ stabilised at the end of the observation time (180 min), the maximum penetration lengths were approximately 42 cm, 50 cm, 56 cm and 66 cm for θ = 0°, 15°, 30° and 45°, respectively. Figure 16 illustrates that T_L_ increased rapidly throughout the initial 20–40 min before achieving a nearly stable condition for all inclination angles. The maximum T_L_ obtained at θ = 45° was approximately 46–68 cm, which was nearly 2.3–3.4 times higher than the corresponding values at θ = 0.0° (about 20 cm). Furthermore, the difference between angles remained consistently significant, with increments of nearly 25–40% observed between θ = 15°, 30°, and 45°.

For scenarios with an underground dam (Fig. 16.b to f), T_L_ increased gradually early in the simulation and reached a steady state when the SWI toe reached the dam. All slope situations show a similar pattern at the beginning, though T_L_ advanced much more quickly on steeper slopes (θ = 45°). There was no noticeable impact of the variations of the underground dam height on T_L_ values, and similar intrusion lengths were observed when varying H_d_ to 7, 9, 11, 13 and 15 cm. The maximum penetration distances were found to be roughly 19 cm, 26 cm, 34 cm and 46 cm for θ = 0°, 15°, 30° and 45°, respectively, as T_L_ stabilised at 15, 20, 30 and 40 min, respectively. The presence of an underground dam blocked SWI, causing the mixing zone to accumulate behind the dam. The mixing zone rose, and the T_L_ values reached a steady state earlier than with the case of no dam (180 min). The SWI toe reached the dam, and the T_L_ values became constant to the end of the simulation for various cases of inclination angle. The presence of the dam had a significant impact to block the intrusion, but variation in the height of the dam had little impact on the T_L_ values.

### Hydrodynamic behaviour of the bottom width of mixing zone (W_1_) in sloping beach coastal aquifers

**Fig. 17:**
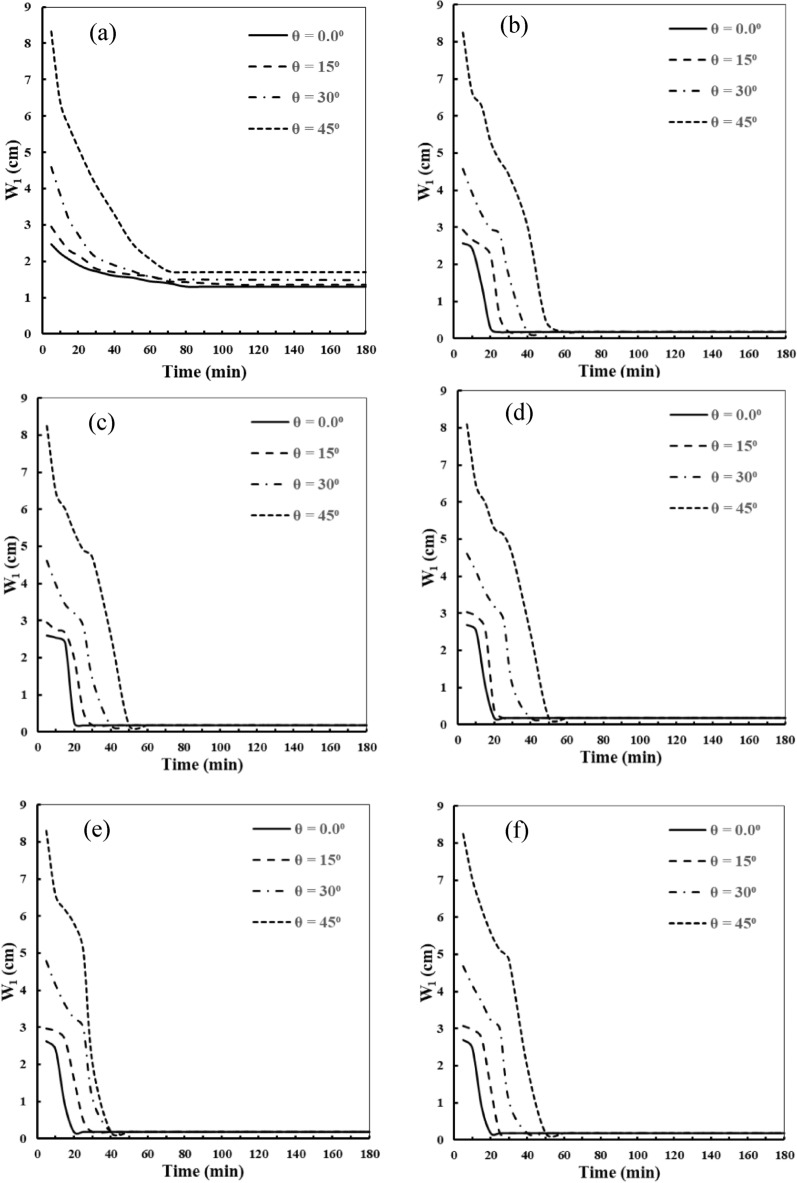
Hydrodynamic behaviour of the bottom width of mixing zone (W_1_) for different values of sloping beach coastal aquifers θ = 0.0°, 15°, 30° and 45°, for different cases of underground dam height (H_d_): (**a**) 0.0 cm, (**b**) 7 cm, (**c**) 9 cm, (**d**)11 cm, (**e**) 13 cm and (**f**) 15 cm.

Figure 17 presents the hydrodynamic behaviour of the bottom width of the mixing zone (W_1_) for different values of sloping beach coastal aquifers (θ) at 0.0°, 15°, 30° and 45°, for various cases of underground dam height (H_d_) at 0.0 cm, 7 cm, 9 cm, 11 cm, 13 cm and 15 cm. The bottom width of the mixing zone exhibited an initial increase in the first 5 min of the simulation, followed by a subsequent decline until a steady state was reached, when the SWI wedge toe arrived at the underground dam location. In addition, the steady state differed with each inclination angle, according to the distance between the seawater boundary and the underground dam. The bottom width of the mixing zone reached a steady state at an earlier time in the vertical boundary beach face compared with the inclined beach faces, where it took a longer time to reach the dam block.

Increasing the inclination angle of the sloping beach face results in an increase in the distance between the boundary and the location of the dam where the dam block starts. As a result, the bottom width of the mixing zone reached a steady state in later stages upon increasing the inclination angle. The values of W_1_ reached a steady state at 20, 30, 40 and 50 min for sloped beach aquifers with angles of 0.0°, 15°, 30° and 45°, respectively. By comparing the results in Fig. 15.a to b, it becomes obvious that increasing the height of the underground dam has a limited effect on the bottom width of the mixing zone. The outcomes of bottom mixing zone width W_1_ remained consistent during the hydrodynamic SWI process over different scenarios of underground dam height. To conclude, increasing the height of the underground dam has a negligible long-term impact on the bottom width of SWI mixing zone. After the SWI wedge toe reached the underground dam bottom, the mixing zone started to rise, and the bottom width W_1_ steadied at a smaller width than in the scenario with no underground dam (Fig. 17.a).

Figure 17 shows that W_1_ declined continuously with time for all inclination beach angles, eventually approaching nearly 0.17 cm after approximately 20–55 min. The initial width W_1_ at θ = 45° reached about 8–8.5 cm, which was nearly three to four times greater than the corresponding values at θ = 0° (approximately 2–3 cm). Furthermore, the complete depletion time increased by nearly 40–60% as the inclination angle increased from 0° to 45°. In the underground dam scenario shown in Fig. 17.a, W_1_ initially showed a gradual drop for all slope conditions, with steeper slopes displaying much larger initial widths. At time zero, W1 exhibited a steep ascent from the lowest to the highest slope angle, with the steepest slope (θ = 45°) measuring almost 8.5 cm and the vertical boundary case (θ = 0°) measuring nearly 2 cm. The bottom mixing zone (W_1_) steadily stabilised after quickly decreasing within the first 60 min. W_1_ achieved near-equilibrium values for all slopes within about 80 min. At equilibrium (120–180 min), W_1_ converged to about 1.4–1.8 cm with less than 20% relative variance among slope conditions, indicating that the impact of slope angle decreased with longer time periods. For Fig. 17.b to f, for the underground dam scenarios with various H_d_ values, W_1_ initially showed a rapid decline for various inclination angles, and higher inclination angles showed a much larger initial mixing zone width. The W_1_ values reached steady state at 20, 30, 40 and 50 min, with the same bottom width W1 equal to 0.17 cm, while the underground dam blocks the intrusion of seawater; this is true for different inclination angles. The final bottom width (W_1_) in the underground dam scenarios is smaller than the cases with no dam. The presence of the dam blocks SWI rapidly and causes the width to decline more rapidly compared with the no-dam scenario. Little impact for varying the dam height is obvious on the values of bottom mixing zone width (W_1_).

### Hydrodynamic behaviour of width of mixing zone (W_2_) at average saturated depth in sloping beach coastal aquifers

**Fig. 18:**
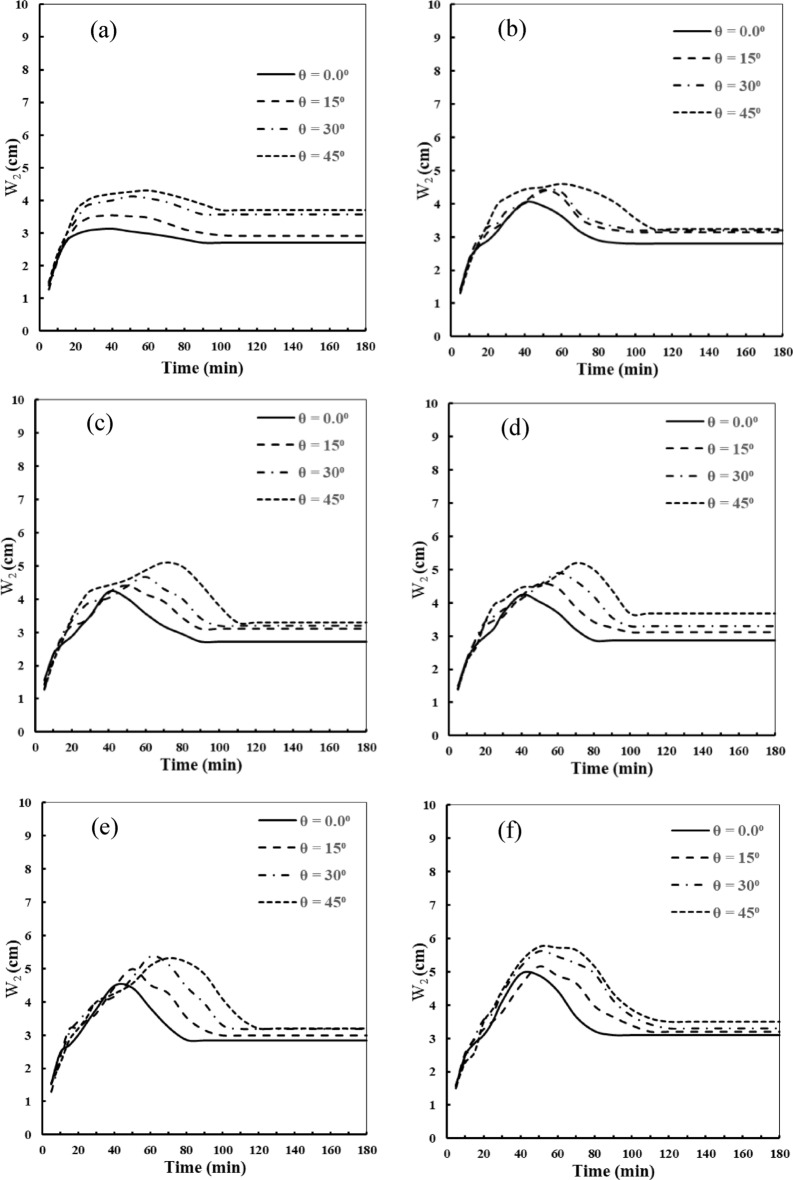
Hydrodynamic behaviour of the mixing zone width (W_2_) at the average saturated depth for different values of sloping beach coastal aquifers θ = 0.0°, 15°, 30° and 45°, for different cases of underground dam height (H_d_): (**a**) 0.0 cm, (**b**) 7 cm, (**c**) 9 cm, (**d**)11 cm, (**e**) 13 cm and (**f**) 15 cm.

Figure 18 presents the hydrodynamic behaviour of the central width of the mixing zone (W_2_) at the average saturated depth for different values of sloping beach coastal aquifers (θ) at 0.0°, 15°, 30° and 45°; and for various cases of underground dam height (H_d_) at 0.0 cm, 7 cm, 9 cm, 11 cm, 13 cm and 15 cm. Increasing the beach inclination angle resulted in a larger increase in the central width of the mixing zone (W_2_) at the mid-aquifer depth than with a vertical boundary aquifer. The inclination beach face angle of 45° exhibited the maximum central mixing zone width (W_2_) compared with the other angle scenarios. In addition, increasing the height of the underground dam had a significant impact on the value of the central width of the mixing zone (W_2_) when compared with the case of no underground dam. In the earlier stage of the simulation, the central width of the mixing zone (W_2_) displayed an initial increase during the first 50 min of the simulation because of slowing the flow close to the underground dam. After the SWI wedge toe reached the dam, the central width of the mixing zone showed a subsequent decline and finally reached the steady state condition after 100 min and was smaller than in the no-underground dam scenario (Fig. 18.a). Increasing the depth of underground dam, as shown in Fig. 16.b to f, led to decline the central width of the mixing zone further than in the shorter underground dam scenarios. To conclude, increasing the angle face of the beach had a significant impact on increasing the central width of the mixing zone; in addition, higher underground dam heights further reduced the central width of the mixing zone, making the central part of the SWI mixing zone narrower.

For Fig. 18.a, in the no-dam scenario, the central mixing zone width (W_2_) at the average saturation depth exhibited a dramatic initial increase over the first half hour in all the slope scenarios. The width peaked early for slopes that are higher. Specifically, W_2_ initially increased by approximately 50% for the highest slope, from approximately 1.5 cm to approximately 3.0 cm for θ = 0° and from approximately 1.5 cm to approximately 4.5 cm for θ = 45°. Following the initial increase, W_2_ showed a slight decline before stabilising after about 80 min. At the conclusion of the simulation (180 min), the steady state W_2_ values for θ = 0°, 15°, 30° and 45° were approximately 2.6 cm, 3.2 cm, 3.5 cm and 3.8 cm, respectively, with a statistically significant increasing trend with increasing slope angle. Figure 18 explains that W_2_ increased rapidly during the first 40–70 min before gradually approaching a steady state. Compared with the vertical beach aquifer (θ = 0°) inclined orientations 15°-45° produced higher peak W_2_ values, with the maximum increase ranging from approximately 12–35%. The highest central width of mixing zone was consistently observed at inclination angles between 30° and 45°, whereas the differences became less pronounced after 100–120 min as the central width of mixing zone approached equilibrium. In Fig. 18.b to f, the values of W_2_ displays a gradual increase over 50, 60, 70 and 80 min for inclination angles (θ) equal to 0°, 15°, 30° and 45°, respectively, for the underground dam scenarios. In addition, W_2_ reached the peak later with higher values than did no-underground dam scenarios. The existence of the underground dam had a significant impact on the expansion of the mixing zone, especially after the SWI wedge toe reached the bottom of the dam. For underground dam height H_d_ equal to 9 cm, the peak values of W_2_ equalled 4.1, 4.3, 4.6 and 5.3 cm for inclination angles (θ) of 0°, 15°, 30° and 45°, respectively. The maximum central mixing zone width (W_2_) equal to 4.9, 5.1, 5.7 and 5.8 cm, was observed for the underground dam height scenario (H_d_) equal to 15cm, with inclination angles of the sloping beach equal to 0°, 15°, 30° and 45°, respectively. The combination of the inclination angle of the sloping beach and the underground dam has a significant impact in creating a wider mixing zone after the SWI toe reached the dam than did case of the no-dam scenario.

### Hydrodynamic behaviour of the mixing zone area (A_m_) at the average saturated depth in sloping beach coastal aquifers

**Fig. 19:**
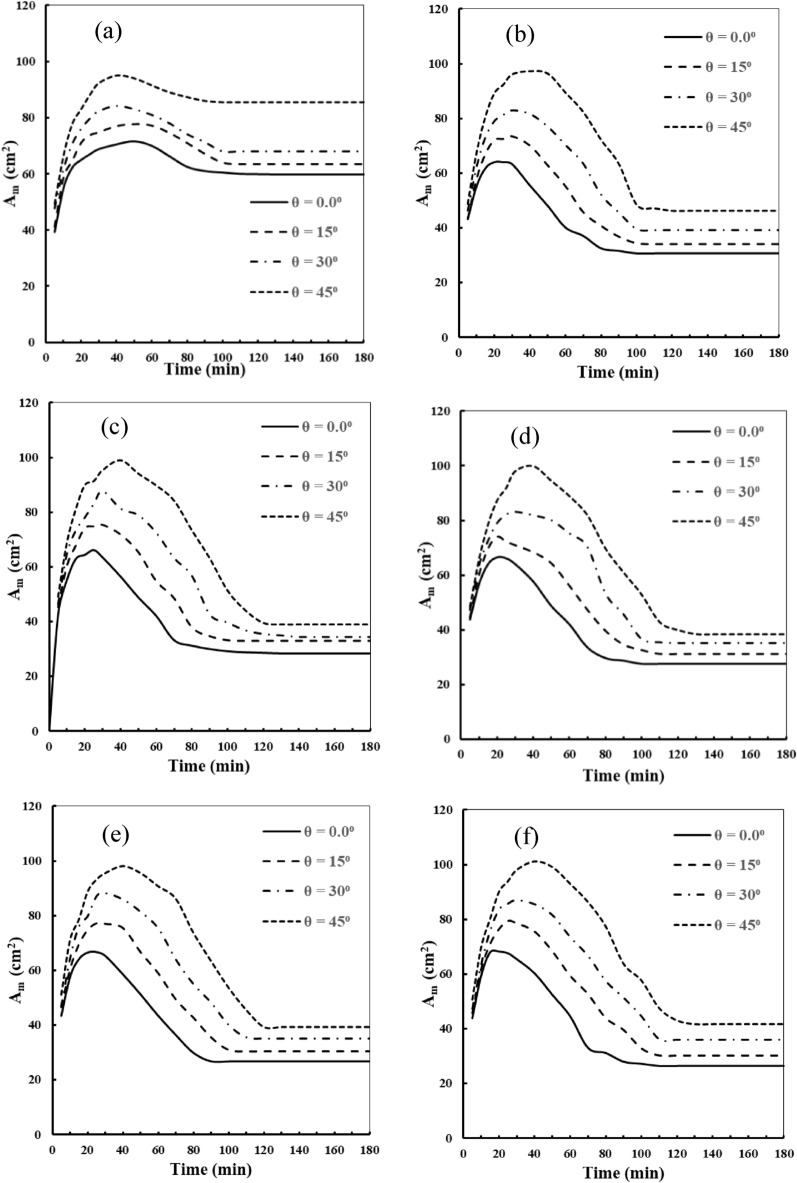
Hydrodynamic behaviour of the mixing zone area (A_m_) for different values of sloping beach coastal aquifers θ = 0.0°, 15°, 30° and 45°, for different cases of underground dam height (H_d_): (**a**) 0.0 cm, (**b**) 7 cm, (**c**) 9 cm, (**d**)11 cm, (**e**) 13 cm and (**f**) 15 cm.

Figure 19 shows the hydrodynamic behaviour of the mixing zone area (A_m_) for different values of sloping beach coastal aquifers (θ) at 0.0°, 15°, 30° and 45°, for various cases of underground dam height (H_d_) of 0.0 cm, 7 cm, 9 cm, 11 cm, 13 cm and 15 cm. The mixing zone area (A_m_) increased in the initial stage of the simulation, as the SWI wedge expanded. After the SWI wedge toe reached the bottom of the underground dam, the dam obstructed the intrusion of seawater inland into the aquifer domain, and the mixing zone area declined until it reached the steady state and ended up lesser than in the no underground dam scenarios. This indicates less overall SWI mixing in the system with a underground dam. Increasing the height of the underground dam led to minimising the area of the SWI mixing zone compared with the case without an underground dam. Increasing the inclination angle of the beach face boundary to 15°, 30° and 45° resulted in an expansion of the mixing zone area more than the vertical boundary case scenario. Increasing the distance between the seawater boundary and the underground dam contributed to extending the dimension of the SWI mixing zone, because of which the area expanded. Increasing the inclination angle resulted in a delay for the SWI mixing area to reach its peak before it again declined after the SWI toe reached the underground dam. The area of the mixing zone reached maximum values at the times of 20, 30, 40 and 50 min for inclination face angles 0.0°, 15°, 30° and 45°, respectively. To conclude, the underground dam does not halt the saltwater intrusion directly; nevertheless, there was a decline in the overall SWI mixing zone area after the wedge reached the dam.

In the no-dam scenario (Fig. 19.a), in the first half hour (A_m_) rapidly increased for all slope conditions, signifying the expansion of the freshwater–saltwater mixing interface. The slope angle and (A_m_) magnitude were positively correlated. During the early phase, the (A_m_) rose from about 40 cm^2^ to 65 cm^2^ for θ = 0°, and from 40 cm^2^ to approximately 96 cm^2^ for θ = 45°, representing a 54% increase between the lowest and highest slope angles. There is a small drop in A_m_ between 40 and 80 min after the peak, after which the region stabilises. During the steady-state period (120–180 min), the mixing area converged to approximately 58 cm^2^, 64 cm^2^, 68 cm^2^ and 88 cm^2^ for θ = 0°, 15°, 30° and 45°, respectively. This implies that the slope affects maintaining a larger mixing zone at equilibrium in a long-term way. Figure 19 demonstrates that the mixing zone area A_m_ increased sharply during the initial stage, reaching peak values within approximately 20–50 min before decreasing toward a steady state condition. Increasing the inclination angle from 0.0° to 45° enhanced the maximum A_m_ values by nearly 20–45%, with the largest difference observed in subfigures b, d, and f. After 100–120 min, the variation among inclination angles decreased considerably, although the 45^o^ condition consistently maintained the highest A_m_ values throughout the simulation period. In scenarios with dams, Fig. 19.b to f, for the cases with underground dam scenarios, the mixing zone area increased gradually and reached peaks equal to 63, 72, 83 and 98 cm^2^ at times 20, 30, 40 and 50 min, for the cases with inclination angles 0°, 15°, 30° and 45°, respectively. After the SWI wedge was blocked by the underground dam, the A_m_ values declined and reached steady state at a smaller area than the case with no-underground dam scenarios. The mixing zone area reached a steady state after 100 min from the beginning, close to the case without an underground dam. The A_m_ ended up with the values equal to 32, 36, 41 and 51 cm^2^ at a steady state for inclination angles 0°, 15°, 30° and 45°, respectively. In addition, increasing the height of the underground dam resulted in more reduction in the SWI mixing zone area at the steady state.

### Hydrodynamic behaviour of the length of the seawater wedge (L_90%_) in sloping beach coastal aquifers

**Fig. 20:**
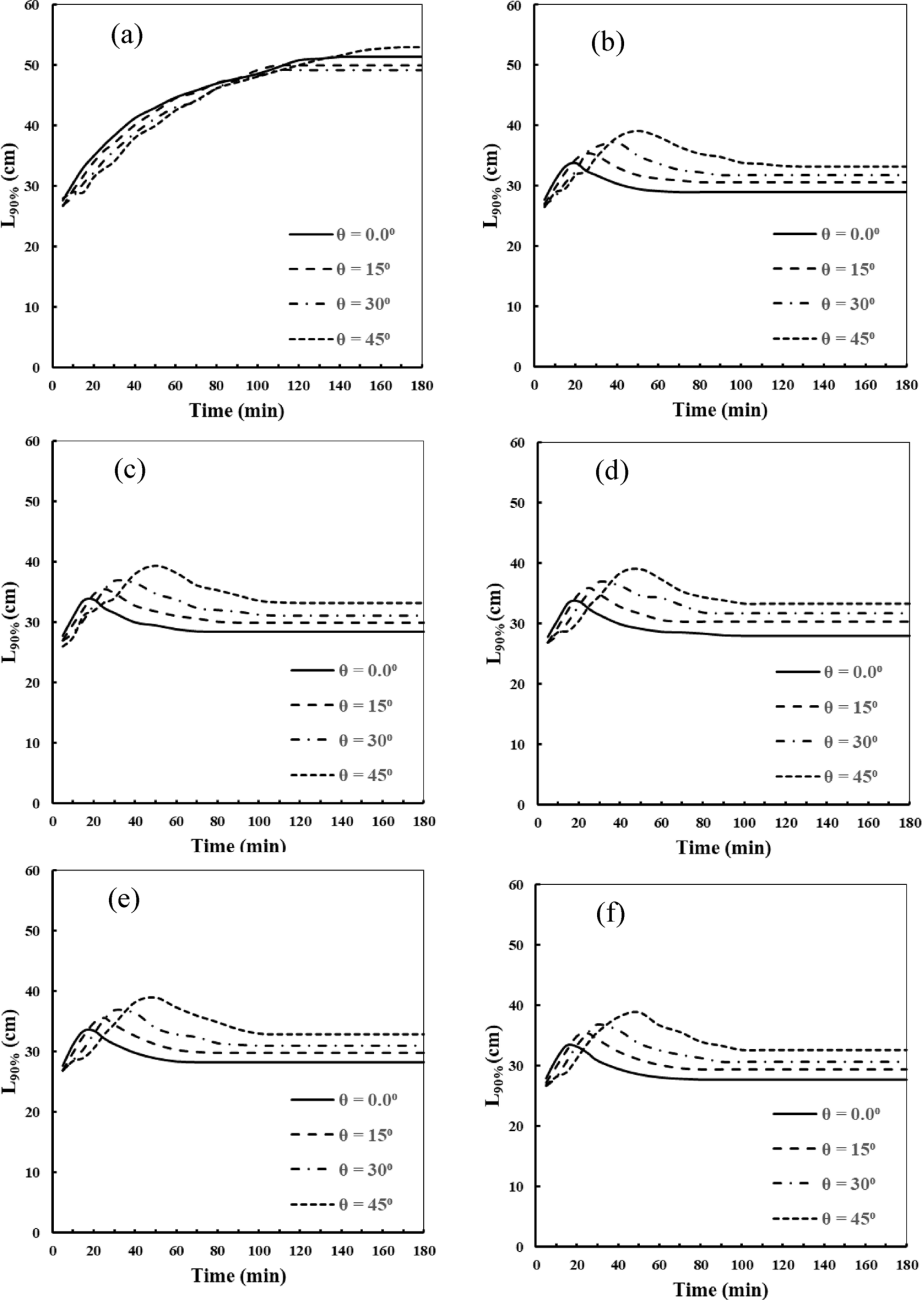
Hydrodynamic behaviour of the length of the seawater wedge (L_90%_) for different values of sloping beach coastal aquifers θ = 0.0°, 15°, 30° and 45°, for different cases of underground dam height (H_d_): (**a**) 0.0 cm, (**b**) 7 cm, (**c**) 9 cm, (**d**)11 cm, (**e**) 13 cm and (**f**) 15 cm.

Figure 20 shows the hydrodynamic behaviour of the length of the seawater wedge (L_90%_) for different values of sloping beach coastal aquifers (θ) at 0.0°, 15°, 30° and 45°, for various cases of underground dam height (H_d_) at 0.0 cm, 7 cm, 9 cm, 11 cm, 13 cm and 15 cm. Figure 18.a shows that the length of the 90% isohaline (L_90%_) increased gradually as the SWI intrusion process occurred until it reached a steady state in the case without a underground dam. In addition, an inclination angle led to a decline in the length of the isohalines (L_90%_) in an earlier stage of the simulation compared with the vertical case scenario, and after continuing the intrusion process, the whole area of the SWI wedge expanded more with inclination than the vertical boundary scenario, and the L_90%_ increased. The height of the underground dam had a significant impact on the length of the isohalines (L_90%_) of the mixing zone. The length of the isohalines (L_90%_) increased in the earlier stage of the simulation and reached the peak when the SWI wedge toe reached the bottom of the underground dam; after that, the length of the isohalines (L_90%_) declined gradually and reached the steady state after 120 min of simulation time. The length of the isohalines (L_90%_) started with lower values in the scenarios of inclination angles 15°, 30° and 45° than the vertical boundary case; after 20 min, the value of L_90%_ increased gradually, with a higher rate in inclination angle scenarios. This is because the inclination of the boundary expands the SWI wedge more than in the no-subsurface barrier scenario, and as a result, the length of the isohalines (L_90%_) exhibited high values. The length of the isohalines (L_90%_) reached their peak value at various times equal to 20, 30, 40 and 50 min from the beginning of the simulation in scenarios with inclination angles 0.0°, 15°, 30° and 45°, respectively for various underground dam heights.

For Fig. 20.a, without an underground dam, L_90%_ steadily rose with time in all slope configurations, indicating the saltwater front’s slow onshore movement. In contrast to a θ = 0° slope, the steeper slopes first showed somewhat lower L_90%_ values from 0 to about 60 min, indicating a delayed progression of high saline levels in steeper profiles. At 60 min, for instance, L_90%_ reached about 42 cm, while for steeper slopes, it remained around 38–40 cm. With only slight variations seen near the end of the simulation, the L_90%_ values for all slopes converge as the run time exceeds 100 min. For θ = 0°, L_90%_ stabilised at 52 cm at 180 min, while the other slopes converged slightly below, ranging between 48 and 50 cm, demonstrating a difference of less than about 8% in all cases. Figure 20 shows that the length of the isohalines (L_90%_) generally increased during the early simulation period and stabilized after approximately 80–120 min. Relative to the vertical beach aquifer 0°, inclined angles (15^o-^45°) increased the peak L_90%_ values by approximately 10–30%, with the highest increments consistently recorded at 45°. The influence of inclination beach face angle became more evident in subfigures (b–f). In the cases with underground dam scenarios, in Fig. 20.b to f, the L_90%_ value reached its peak rapidly, with values of 34, 35, 37 and 40 cm for inclination angles 0.0°, 15°, 30° and 45°, respectively. The installation of an underground dam had a significant impact to block the intrusion of seawater and reduce the values of L_90%_. In the case with no dam, the seawater intruded into the aquifer without any obstruction, so a gradual increase in L_90%_ was seen until the end of the simulation. On the other hand, in dam-scenarios, the seawater was blocked by the dam, and the mixing zone started to rise, meaning the L_90%_ values declined slowly to the end, with smaller values after 100 min compared with no-dam scenarios. The steady state values of L_90%_ equalled 32, 33, 34 and 35 cm for inclination angles equal to 0.0, 15, 30 and 45, respectively. Increasing the height of the dam had little impact on the values of L_90%_.

### Hydrodynamic behaviour of average mixing zone width (MZW) at average saturated depth in sloping beach coastal aquifers

**Fig. 21:**
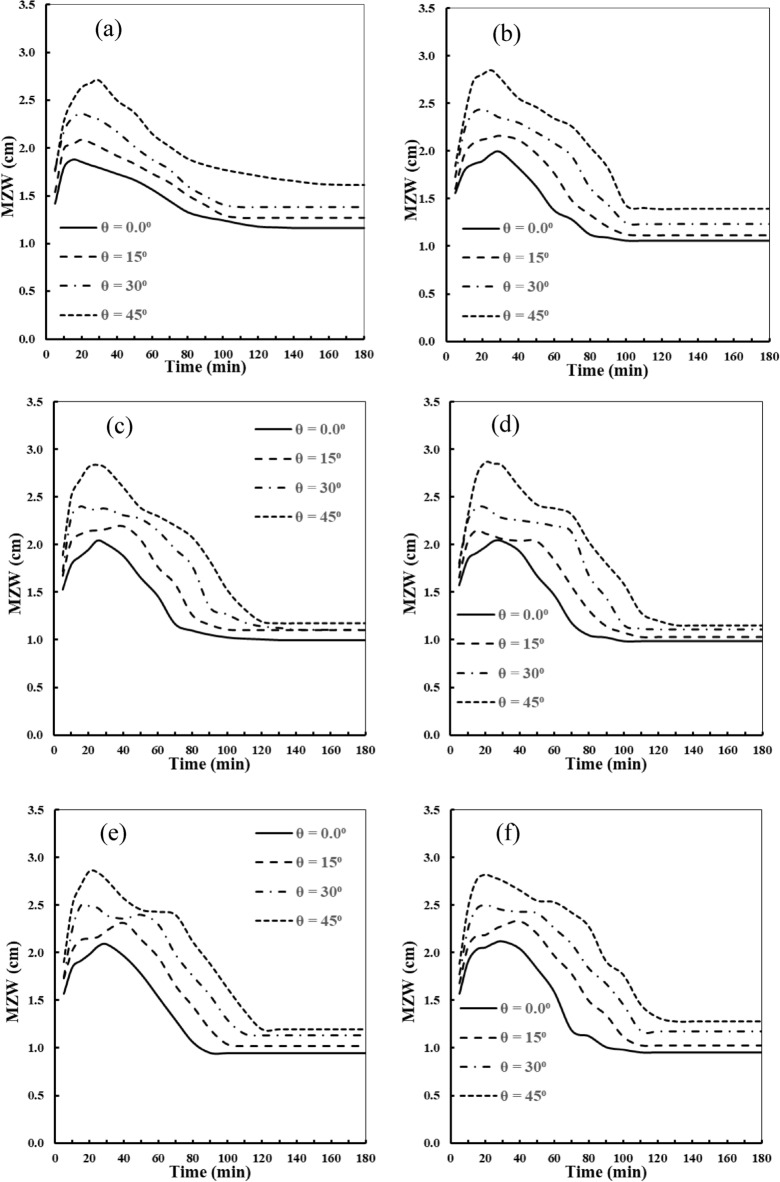
Hydrodynamic behaviour of average mixing zone width (MZW) for different values of sloping beach coastal aquifers of θ = 0.0°, 15°, 30° and 45°, for different cases of underground dam height (H_d_): (**a**) 0.0 cm, (**b**) 7 cm, (**c**) 9 cm, (**d**)11 cm, (**e**) 13 cm and (**f**) 15 cm.

Figure 21 shows the hydrodynamic behaviour of the average mixing zone width (MZW) for various values of sloping beach coastal aquifers (θ) at 0.0°, 15°, 30° and 45°, for different cases of underground dam height (H_d_) at 0.0 cm, 7 cm, 9 cm, 11 cm, 13 cm and 15 cm. The value of the average mixing zone width (MZW) equals the area of the mixing zone divided by the length of the isohalines L_90%_. Increasing the mixing zone area produced a higher mixing zone width. Increasing the inclination angle of the beach slope face resulted in an increase the average MZW for various heights of an underground dam. The inclination of boundary provides the expansion of the mixing zone; the mixing zone area increased, and as a result the MZW also increased. The inclination angle of beach face led to a widening of the mixing zone, and the average MZW increased. Early in the simulation, the MZW exhibited a rapid increase, and the value of MZW reached the peak before 40 min. Once the SWI wedge toe was obstructed by the underground dam, the MZW value declined and stabilised below the no-underground dam scenario. For various underground dam heights, the MZW reached a steady state 120 min after the start of the simulation. Overall, the presence of underground dams resulted in a narrower SWI mixing zone at the steady state condition.

For Fig. 21.a, without an underground dam, the average mixing zone width (MZW) initially rose quickly, peaking for all slope angles during the first half hour. The peak MZW showed a positive correlation with the beach slope, increasing by around 66% with slope steepness, from about 1.8 cm for θ = 0° to nearly 2.8 cm for θ = 45°. The MZW peaked and then steadily dropped for about an hour before levelling off. In addition, the MZW stabilised at lower levels throughout the steady-state phase (120–180 min), around 1.2 cm, 1.3 cm, 1.5 cm and 1.7 cm for θ = 0°, 15°, 30° and 45°, respectively. Steeper slopes continually maintained a wider mixing zone than flatter slopes, even though the values were reduced from the peak values. Figure 21 illustrates that the average mixing zone width (MZW) increased rapidly during the first 20–50 min, followed by a gradual decline toward stable values after approximately 100 min. Increasing the inclination angle from 0° to 45^o^ enhanced the peak MZW values by nearly 15–40%, with the greatest increases observed in subfigures (b), (c), and (d). Although all simulations exhibited a decreasing trend at later times, the inclined conditions consistently maintained higher MZW values than the vertical beach configuration (θ = 0°) throughout the simulation duration. In scenarios with an underground dam, as presented in Fig. 21.b to f, the inclination of the beach face of the sea boundary resulted in a widening of the mixing zone compared with the vertical beach boundary. The MZW increased rapidly and reached a peak at values of 1.8, 2.2, 2.4 and 2.7 at inclination angles equal to 0.0°, 15°, 30° and 45°, respectively, for various heights scenarios of the underground dam. The values of the MZW with underground dam scenarios, at their peak, are a little larger than the case without a dam. After the peak, at 20 min, the MZW values exhibited a gradual decline, ultimately ending up with smaller values compared with the no-dam scenarios at 120 min to the end of simulation by 180 min. The steady state values of the MZW equalled 1.1, 1.2, 1.4 and 1.5 cm with inclination angles equal to 0.0°, 15°, 30° and 45°, respectively, and were narrower than the scenarios without an underground dam.

### Impact of mechanical dispersion on seawater-freshwater mixing zone

**Fig. 22:**
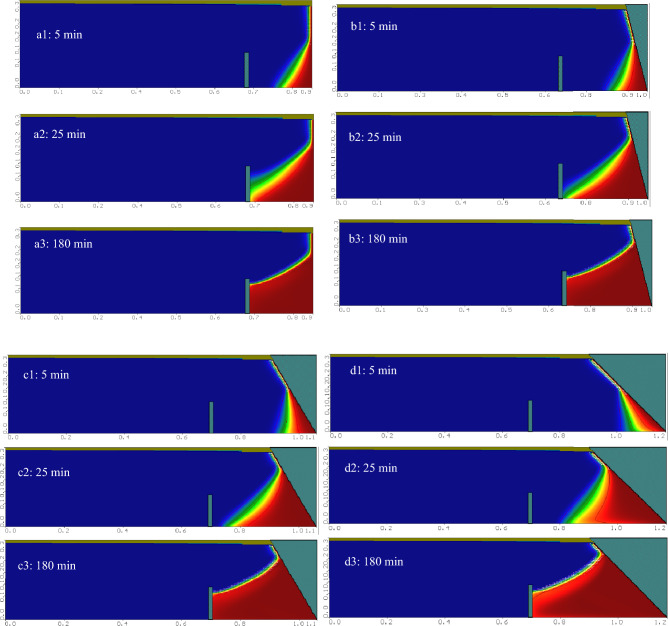
Steady state seawater intrusion for sloped beach coastal aquifers for the case with underground dam (11 cm depth), for increasing longitudinal despersivity (5α_L_) for various inclination angles of the beach face: (**a**) 0.0°, (**b**) 15°, (**c**) 30° and (**d**) 45°; the black lines indicate the 10, 50 and 90% isohalines of the mixing zone.

Figure 22 presents the steady-state saltwater intrusion in sloped beach coastal aquifers with the case of underground dam at 11 cm depth when the value of longitudinal dispersivity is increased five times (5α_L_). At 0° sloped beach case, higher values of longitudinal dispersivity widens the mixing zone and smooths the seawater intrusion wedge interface. The 10% isohaline moves further inland representing stronger longitudinal seawater dispersion. At 15°, the seawater intrusion wedge develops longer and more curved. Increased dispersivity in the longitudinal direction enlarges the seawater-freshwater mixing zone around the underground dam and causes more diffuse salinity contours, demonstrating increased salt transfer along the flow direction. At 30° sloped beach case, the seawater-freshwater mixing zone expands further, with larger width, specifically close the saline intrusion wedge. The inclined beach of coastal aquifer promotes groundwater circulation and stronger longitudinal dispersion, producing a thicker transition zone between seawater and freshwater.

The steepest sloped beach case (45°) demonstrates the greatest seawater-freshwater mixing zone and seawater dispersion. Isohalines stretch along the slope, and mixing zone becomes wider. While the underground dam still limits the total saltwater intrusion penetration, the combined effects of steep beach slope and high values of longitudinal dispersivity significantly enhance the inland salinity diffusion. Overall, increasing the value of longitudinal dispersivity greatly expands the seawater-freshwater mixing zone, particularly at higher values of sloped coastal beach (30°, and 45°). Compared with transverse dispersivity, longitudinal dispersivity has a stronger impact on the saltwater intrusion in sloping coastal aquifers with underground dams.

**Fig. 23:**
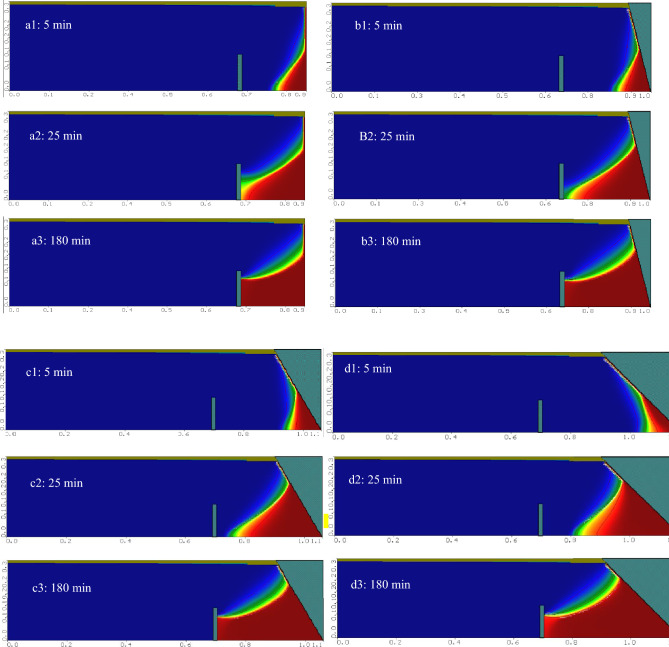
Steady state seawater intrusion for sloped beach coastal aquifers for the case with underground dam (11 cm depth), for increasing transverse despersivity (5α_T_) for various inclination angles of the beach face: (**a**) 0.0°, (**b**) 15°, (**c**) 30° and (**d**) 45°; the black lines indicate the 10, 50 and 90% isohalines of the mixing zone.

Figure 23 demonstrates the steady-state saltwater intrusion in sloped beach coastal aquifers with an underground dam at 11 cm depth when the transverse dispersivity (α_T_) increased 5 times to (5α_T_). At (0°) sloped beach case, the saltwater intrusion wedge remains compact with a narrow transition zone near the seawater intrusion wedge toe. Increasing the value of transverse dispersivity slightly smooths the seawater wedge interface, producing a broader but still symmetric mixing region around the underground dam. At 15°, the seawater-freshwater mixing zone expands upward along the sloping beach face. The inclined boundary changes the groundwater flow and enhances the lateral saltwater transport, causing the isohalines of concentration to become more curved and vertically stretched. The underground dam still restricts inland saltwater movement, while the seawater-freshwater interface around the dam thickens because of stronger transverse mixing.

For the 30° slope, the mixing zone becomes wider and smoother, with greater separation between the 50% and 90% isohalines close to the underground dam. The sloping geometry increases the shear effects along the saline wedge boundary, enhancing the dispersion and the dilution processes. The 45° slope shows the largest and most diffuse mixing zone. Isohalines are strongly elongated and widely separated, reflecting substantial transverse dispersion. The combination of steep slope beach and the underground dam strengthen shear-driven mixing, particularly close to the upper seawater intrusion wedge, while the underground dam still restricts the inland intrusion. Overall, increasing beach slope strengthens the impact of transverse dispersivity by enlarging and thickening the seawater-freshwater mixing zone. While the underground dam efficiently controls seawater penetration, both slope geometry and transverse dispersion play important roles in seawater intrusion behavior in sloping coastal aquifers.

### Impact of mechanical dispersion on seawater-freshwater mixing zone

**Fig. 24:**
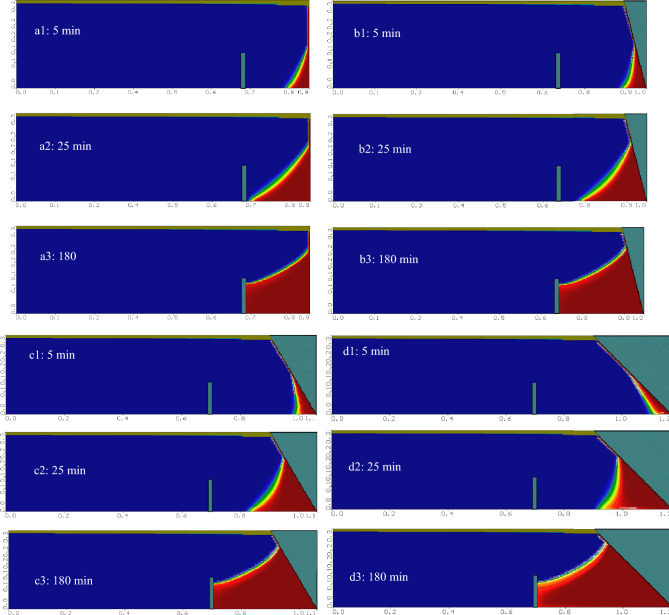
Steady state seawater intrusion for sloped beach coastal aquifers for the case with underground dam (11 cm depth), for minmizing hydraulic conductivity (0.5K) for various inclination angles of the beach face: (**a**) 0.0°, (**b**) 15°, (**c**) 30° and (**d**) 45°; the black lines indicate the 10, 50 and 90% isohalines of the mixing zone.

Figure 24 displays the steady-state saltwater intrusion in sloped beach coastal aquifers with an underground dam at 11 cm depth under reduction of the hydraulic conductivity value (0.5 K). At 0° slope, decreased hydraulic conductivity value suppresses the saltwater movement and limits inland seawater intrusion. The isohalines remain compact and closely spaced, indicating a narrow transition zone between seawater and freshwater. At 15° slope, the sweater intrusion wedge becomes slightly elongated along the sloping shoreline, but lower value of hydraulic conductivity still restricts the groundwater flow and the inland advancement of saltwater. The seawater-freshwater mixing zone expands only moderately close to the seawater intrusion wedge toe. At 30° slope, the seawater-freshwater interface curves upward along the beach face, although the seawater-freshwater mixing zone remains relatively confined. The underground dam continues to block inland salt transport and supports freshwater accumulation behind the dam.

The steepest beach face slope 45° delivers the greatest upward stretching of the saline intrusion wedge because of the inclined boundary. However, the reduced value of hydraulic conductivity limits seawater intrusion penetration and prevents major widening of the seawater-freshwater mixing zone. Though the isohalines of salt concentration become more curved and vertically extended, the seawater-freshwater transition zone remains thinner because the groundwater circulation and the solute transport are weaker. Overall, decreasing the value of hydraulic conductivity reduces the saltwater intrusion intensity and compresses the seawater-freshwater mixing zone in sloped beach coastal aquifers. While higher values of beach slopes promote the upward dispersion of salinity, lower hydraulic conductivity and the underground dam effectively restrict inland seawater migration and protect freshwater resources.

**Fig. 25:**
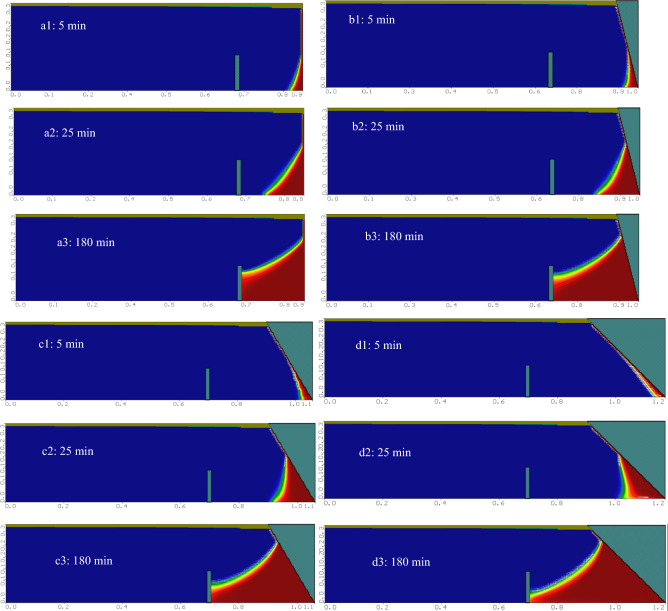
Steady state seawater intrusion for sloped beach coastal aquifers for the case with underground dam (11 cm depth), for minmizing hydraulic conductivity (0.25K) for various inclination angles of the beach face: (**a**) 0.0°, (**b**) 15°, (**c**) 30° and (**d**) 45°; the black lines indicate the 10, 50 and 90% isohalines of the mixing zone.

Figure 25 displays the steady-state saltwater intrusion in sloped beach coastal aquifers with an underground dam at 11 cm depth under strongly reduced hydraulic conductivity conditions (0.25 K). At 0° slope, the strong reduction of the hydraulic conductivity value (0.25 K) substantially suppresses saltwater movement and produces a very compact seawater intrusion wedge close to the coastal beach. The isohalines of salt concentration remain tightly grouped, representing a thinner seawater-freshwater mixing zone than in the 0.5 K case, while the underground dam effectively maintains a stable freshwater region landward of the barrier. At 15° slope, the seawater intrusion wedge becomes slightly elongated along the sloping beach face, but inland saltwater advancement remains strongly limited. Compared with the 0.5 K case, the seawater intrusion wedge toe is shorter, and the seawater-freshwater transition zone is narrower, reflecting weaker groundwater flow and reduced solute transport. The isohalines of salt concentration remain closely spaced, indicating minimal spreading.

At 30° slope, the seawater-freshwater interface curves upward along the beach face, but the mixing zone remains well confined. The underground dam continues to block inland salt transport, and compared with higher permeability cases, the salinity contours are sharper and less diffuse, showing reduced spreading. The steepest slope (45°) displays the strongest upward extension of the seawater intrusion wedge along the inclined beach line. However, even under this geometry, the 0.25 K condition greatly limits seawater intrusion penetration and keeps the mixing zone thinner than in the 0.5 K case. The isohalines are more compressed, indicating reduced groundwater circulation and weaker mixing despite the steep slope. Overall, reducing the value of hydraulic conductivity from 0.5 K to 0.25 K further reduces saltwater intrusion intensity and substantially compresses the seawater-freshwater mixing zone in all slope tested cases. Although increasing the beach-faced inclination still promotes upward salinity spreading, the very low hydraulic conductivity and the underground dam dominate the system response by strongly limiting inland seawater intrusion and enhancing freshwater protection.

### Groundwater velocity plot

**Fig. 26:**
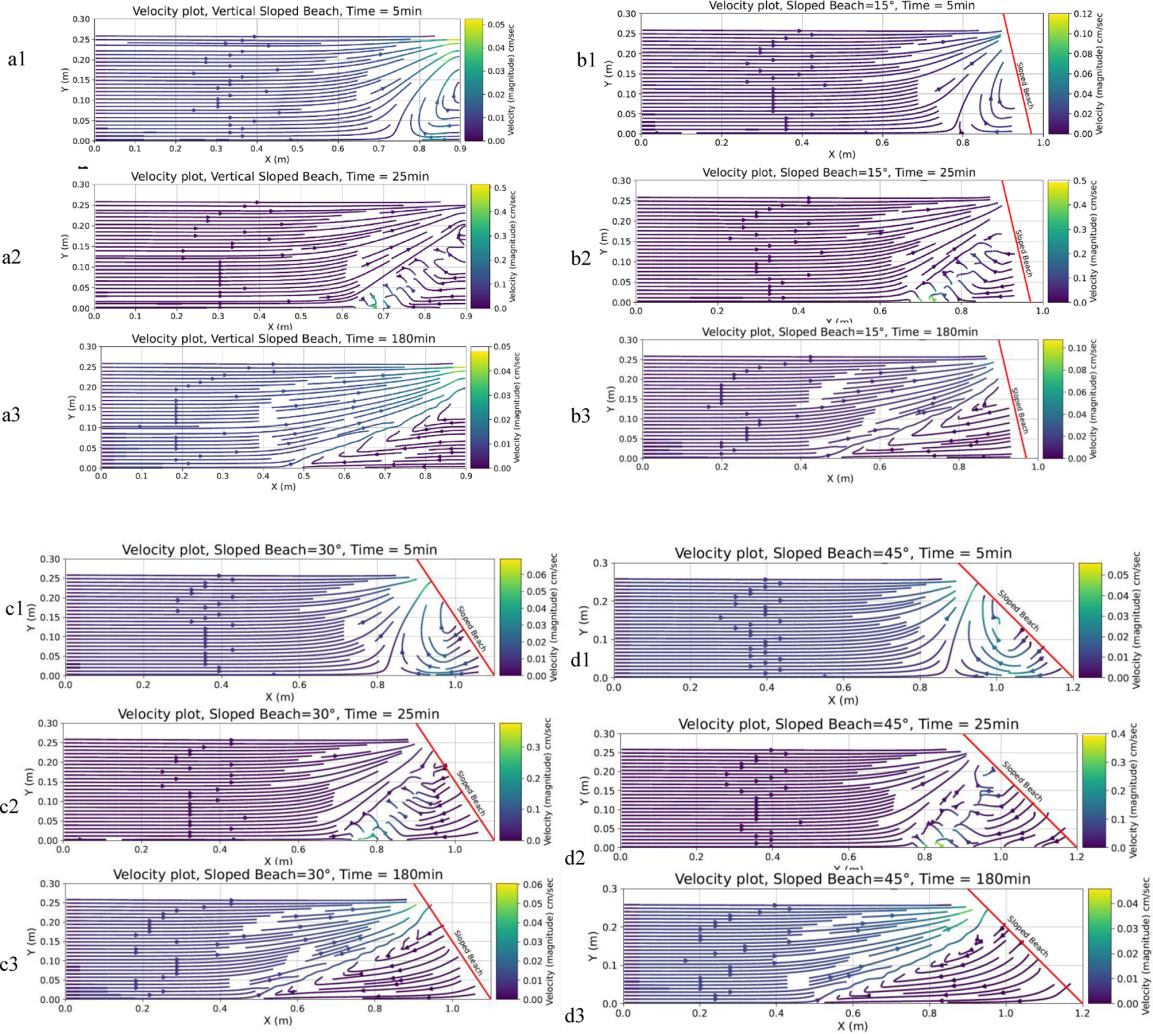
Velocity plot for various sloped beach coastal aquifers: (**a**) 0.0°, (**b**) 15°, (**c**) 30°, and (**d**) 45° at various times equals 5, 25, and 180 min.

Figures 26 illustrates the temporal evolution of groundwater flow velocity fields for saltwater intrusion in sloping beach coastal aquifers with four various beach cases were 0.0°, 15°, 30°, and 45°, it was assessed at 5, 25, and 180 min. At 5 min, the groundwater flow velocities remain low and uniform in the freshwater zone, with maximum groundwater velocity values equals 0.05, 0.12, 0.06, and 0.05 cm/sec for sloping beach equal 0.0°, 15°, 30°, and 45°, respectively. The flow lines are mostly horizontal in the freshwater region and begin to be curved near the coastal line, representing the initial establishment of the seawater intrusion wedge. The case of sloping beaches 15°, 30°, and 45° display smoother flow transitions and wider circulations areas in comparison with the steep seawater-freshwater interface found in the case of vertical beach coastal aquifer case.

At 25 min, the impact of variable density-driven flow becomes more pronounced as the saltwater wedge intrudes more inland. The magnitudes of groundwater flow velocity increase significantly near to the seawater intrusion wedge toe, approximately equals 0.5, 0.5, 0.3, and 0.45 cm/sec for sloping beaches equal 0.0°, 15°, 30°, and 45°, respectively. The flow lines become increasingly close to the seawater coastal boundary, reflecting the increased mixing between seawater and freshwater and recirculation process. Steeper sloping beach coastal cases show broader flow dispersion along the coastal beach face; on the other hand, the vertical beach case maintains a more concentrated seawater intrusion front. After 180 min, the groundwater flow system and the seawater intrusion processes reached a steady-state condition characterized by stronger curvature of the flow lines close to the seawater-freshwater wedge. Maximum groundwater velocity declines again to approximately 0.05, 0.10, 0.03, and 0.05 cm/sec for the inclination beach coastal cases equal 0.0°, 15°, 30°, and 45°, respectively. The coastal aquifer with 45° beach face displays the greatest seawater intrusion extent and the widest seawater-freshwater mixing zone, on the other hand the vertical coastal beach case presents the most confined saline interface. Overall, the results describe that increasing the beach slopes face enhances the lateral seawater intrusion process and changes the groundwater flow velocity distribution over time.

**Fig. 27:**
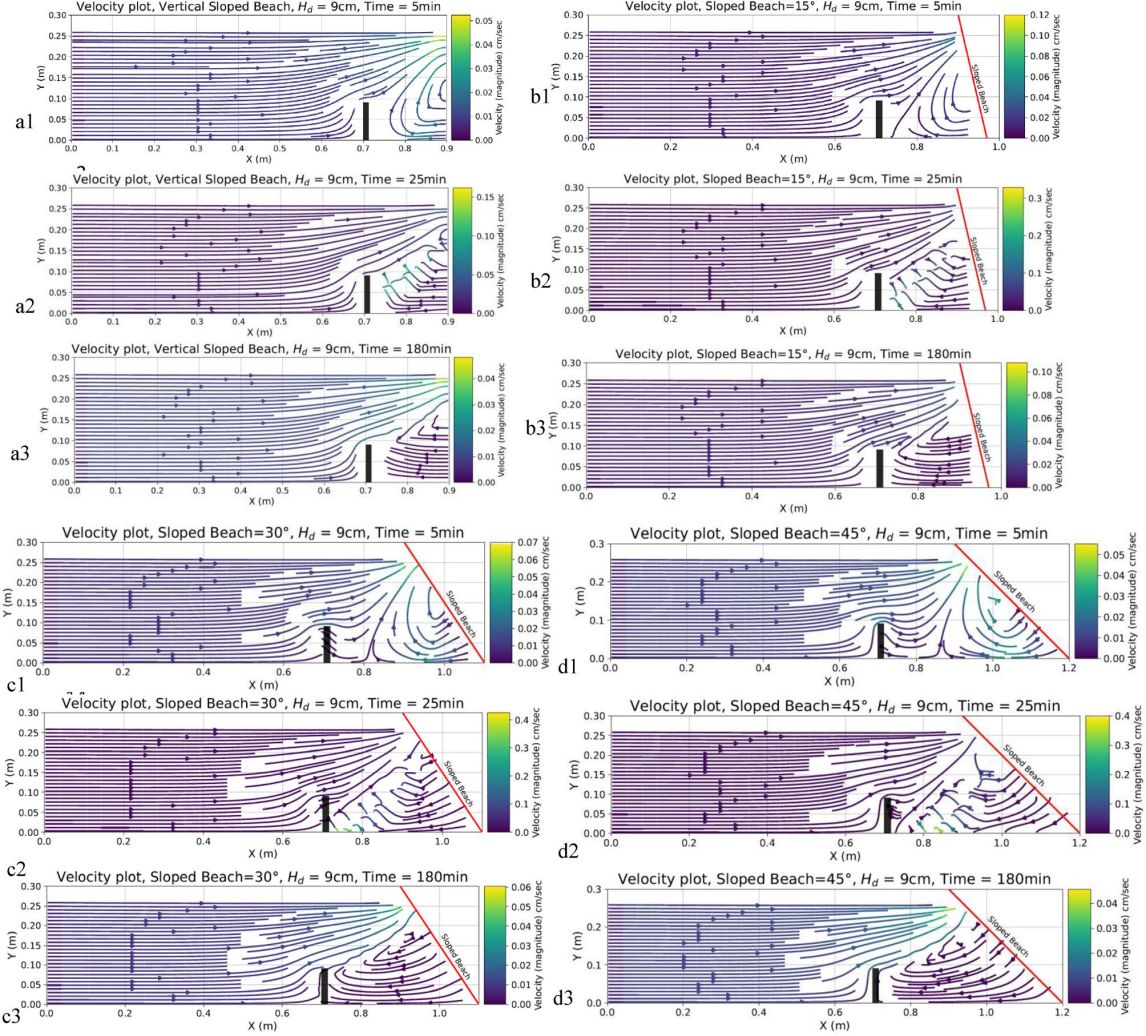
Velocity plot for the case of underground dam depth equals 9 cm for various sloped beach coastal aquifers: (**a**) 0.0°, (**b**) 15°, (**c**) 30°, and (**d**) 45° at various times equals 5, 25, and 180 min.

Figure 27 shows the variation of the groundwater flow velocity for the case of underground dam with depth 9.0 cm, for four various beach slopes of coastal aquifer were 0.0°, 15°, 30°, and 45°, evaluated at times of 5, 25, and 180 min. At 5 min, the underground dam starts to disturb the normal flow direction, while the groundwater flow velocity field remains relatively uniform in the freshwater zone. Maximum observed groundwater velocity values are roughly 0.05 cm/sec for the vertical coastline, 0.12 cm/sec for the 15° slope, 0.07 cm/sec for the 30° slope, and 0.05 cm/sec for the 45° slope. In contrast to Fig. 26, the flow lines in Fig. 27 are redirected around the underground dam, resulting in the creation of localized circulation zones, mixing between seawater and freshwater, and a decline in the direct seawater intrusion movement inland into coastal aquifer.

After 25 min, the impact of the underground dam develops more pronounced as the flow lines bend sharply close to the barrier. Maximum groundwater flow velocities increase to approximately 0.15, 0.30, 0.40, and 0.45 cm/sec for the 0°, 15°, 30°, and 45° slopes, respectively. The underground dam concentrates the flow close to its crest and restricts the inland extension of the seawater intrusion wedge. After 180 min, the groundwater flow system reached steady state condition with minimized groundwater velocity magnitudes of approximately 0.04 – 0.06 cm/sec for all tested slope beach cases. The front of the saltwater intrusion remains confined close to the upstream side of the underground dam, showing the impact of underground dam to limit the seawater intrusion process. In comparison with Fig. 26, the seawater intrusion wedge length and the seawater-freshwater mixing zone are noticeably smaller, on the other hand the groundwater flow field becomes more concentrated around the underground dam. Overall, the results indicate that the underground dam significantly modifies the groundwater flow behaviour, declines seawater intrusion penetration, and improves hydraulic resistance against seawater intrusion, especially for the cases of steeper beach slopes.

The current research extends the published work of Chang et al.^36^ on seawater intrusion in the presence of underground dam by incorporating the impacts of sloping beach coastal aquifers and underground dam height (H_d_). Both studies demonstrated that underground dams do not immediately affect seawater intrusion during the early stages, and that substantial blocking impacts appear only after the seawater intrusion wedge toe reaches the dam position. In both investigations, the seawater-freshwater mixing zone initially widened, specifically the central width (W_2_) and mean mixing-zone width (MZW), before shrinking once the dam efficiently restricted saline water migration. Whereas Chang et al.^36^ focused mainly on the impacts of dispersivity and barrier geometry, the present study demonstrated that aquifer inclination angle (θ) strongly controls mixing-zone dynamics. Increasing θ from 0.0° to 45° enlarged the bottom width (W_1_) central width (W_2_), and isohaline length (L_90%_) during transient conditions, while higher H_d_ reduced W_2_ and subsurface groundwater discharge (SGD) with only minor impacts on toe length (L_toe_) and W_1_. In addition, steeper slopes delayed the arrival of the seawater intrusion wedge to the dam because of the increased travel distance. Therefore, the present study advances previous research by demonstrating the coupled influence of aquifer slope and underground dam configuration on seawater intrusion hydrodynamics and mixing-zone evolution in coastal aquifers.

The outcomes of the current study are limited for shallow unconfined coastal aquifer with sloped beach face ranged from 0.0° to 45°, and with underground dam ratio depth (H_d_/h_s_) ranges from 0.25 to 0.58. This study investigated the influences of underground dams on the hydrodynamic behavior of the saltwater-freshwater mixing zone in sloping beach coastal aquifers using numerical simulations through SEAWAT code but delivers limited physical interpretation of the governing processes. Physically, steeper slopes increase hydraulic gradients and flow velocities, enhancing shear at the seawater–freshwater interface and promoting stronger dispersion, which widens the mixing zone. Underground dams further reshape these patterns by redirecting groundwater flow, creating slower zones upstream and faster pathways over or around the structure. However, the study is based on a 2D homogeneous modeling framework and does not account for aquifer heterogeneity or tidal fluctuations, which may significantly influence real coastal systems by generating more complex flow variability and salinity dynamics.

## Conclusion

The current study investigated the hydrodynamic behaviour of seawater-freshwater mixing zone in sloping beach coastal aquifers under different underground dam heights and beach inclination angles. The numerical simulations using SEAWAT model demonstrated that both aquifer geometry and underground dam configuration significantly influence the temporal evolution and spatial extent of seawater intrusion.

The results display that underground dams did not immediately reduce seawater intrusion during the early stages of numerical simulation. Noticeable impacts observed only after the seawater intrusion wedge toe arrived the dam location. At that stage, the underground dam restricted inland seawater intrusion, causing the seawater-freshwater mixing zone to rise vertically and gradually shrink toward a smaller steady-state condition than in no-dam scenarios. The presence of underground dams reduced the steady-state mixing-zone area from approximately 58–88 cm^2^ in no-dam conditions to nearly 32–51 cm^2^ depending on beach slope condition. Increasing the underground dam height also reduced the central mixing-zone width and subsurface groundwater discharge, while exerting only limited influence on the bottom mixing-zone width and seawater intrusion length.

Beach inclination angles had a substantial impact on seawater intrusion dynamics. Increasing the slope angle from 0° to 45° widened the mixing zone and increased seawater intrusion penetration. The seawater intrusion length increased from nearly 42 cm at θ = 0° to approximately 66 cm at θ = 45° under no-dam conditions. Similarly, the initial bottom mixing-zone width increased from about 2 cm to approximately 8.5 cm as the slope angle increased. Steeper slopes also delayed the arrival of the seawater intrusion wedge at the underground dam, causing steady-state conditions to occur later. In addition, the peak average mixing-zone width increased by nearly 66% between θ = 0° and θ = 45°.

The combined influence of steep beach slopes and underground dams produced temporary enlargement of the mixing zone during the transient stage, followed by a clear reduction after the dam began blocking the seawater intrusion. Although underground dams effectively reduced the long-term extent of seawater intrusion, their performance depended strongly on both dam height and coastal aquifer geometry. In conclusion, this study provides valuable insights into the role of aquifer geometry in managing saltwater intrusion. The results indicate that both the slope of the coastal aquifer and the underground dam depth substantially control the temporal evolution and spatial extent of the seawater–freshwater mixing zone. The development of efficient methods to control SWI in coastal aquifers, especially in areas with different slope characteristics and constructed underground barriers, depends on these outcomes.

From a practical perspective, the findings emphasize the importance of incorporating aquifer beach slope and underground dam dimensions into coastal groundwater management strategies. Properly designed underground dams can significantly reduce seawater intrusion and improve the long-term sustainability of freshwater resources in coastal aquifers. Future studies should consider heterogeneous aquifer properties, tidal fluctuations, variable recharge conditions, and sea-level rise to better simulate real coastal environments. Additional laboratory experiments and three-dimensional numerical modelling are also recommended to further improve understanding of seawater intrusion processes in complex coastal systems.

## Data Availability

The data is available on request to the corresponding author.
